# Role of subduction dynamics on the unevenly distributed volcanism at the Middle American subduction system

**DOI:** 10.1038/s41598-023-41740-y

**Published:** 2023-09-07

**Authors:** Meng Liu, Haiying Gao

**Affiliations:** Department of Earth, Geographic, and Climate Sciences, University of Massachusetts Amherst, 627 North Pleasant St., Amherst, MA 01003 USA

**Keywords:** Tectonics, Seismology

## Abstract

A typical subduction of an oceanic plate beneath a continent is expected to be accompanied by arc volcanoes along the convergent margin. However, subduction of the Cocos plate at the Middle American subduction system has resulted in an uneven distribution of magmatism/volcanism along strike. Here we construct a new three-dimensional shear-wave velocity model of the entire Middle American subduction system, using full-wave ambient noise tomography. Our model reveals significant variations of the oceanic plates along strike and down dip, in correspondence with either weakened or broken slabs after subduction. The northern and southern segments of the Cocos plate, including the Mexican flat slab subduction, are well imaged as high-velocity features, where a low-velocity mantle wedge exists and demonstrate a strong correlation with the arc volcanoes. Subduction of the central Cocos plate encounters a thick high-velocity feature beneath North America, which hinders the formation of a typical low-velocity mantle wedge and arc volcanoes. We suggest that the presence of slab tearing at both edges of the Mexican flat slab has been modifying the mantle flows, resulting in the unusual arc volcanism.

## Introduction

Subduction plays a major role in driving plate tectonics on earth and controls many fundamental tectonic processes. Subduction zones can host great earthquakes, volcanic eruptions, and tsunamis, all of which have significant impacts on natural systems and society. An important characteristic of subduction zones is the observed along-strike variation in a wide variety of properties and processes. For example, significant along-strike variations in arc volcanism/magmatism, seismicity, and fault-slip behaviors have been observed along the subduction margins. The subduction margins also vary substantially along strike in terms of the age and geometry of the subducting plate, the plate convergence rate, crustal thickness of the overriding plate, the composition of the arc volcanoes, and distribution of subducting topography. It is commonly agreed that the geometry variation of the subducting oceanic plates plays a critical role in controlling the nonuniform distribution of arc volcanoes along the subduction margins. However, the spatial distribution patterns of arc magmatism/volcanism cannot be solely explained by the slab geometry.

**Figure 1:**
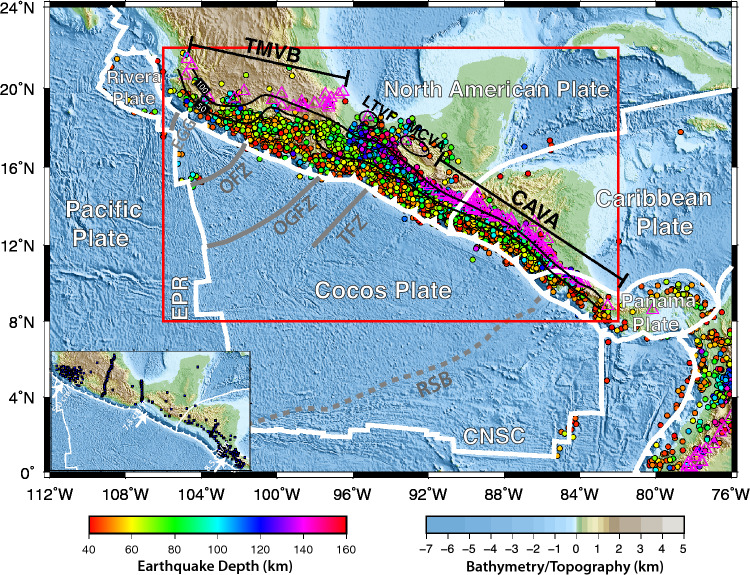
Tectonic setting of the Middle American subduction system. The thick white lines mark the major plate boundaries. The color-coded dots denote the earthquakes with magnitudes >  = 3.0 between 1997 and 2018 (https://earthquake.usgs.gov/data/comcat). The magenta triangles denote the Quaternary volcanoes. The black contours represent the plate interfaces at the depths of 50 km and 100 km extracted from Slab2.0^11^. The gray dashed line represents the rough-smooth boundary (RSB^6^). The red box shows the imaging area in Figs. 2, 3, 4. The inset map shows the seismic stations (blue squares) used in this study and the convergence rates of the Cocos plate along the trench^4^. *ERP* the East Pacific Rise, *CNSC* the Cocos-Nazca spreading center, *EGG* the El Gordo Graben, *OFZ* the Orozco fracture zone, *OGFZ* the O’Gorman fracture zone, *TFZ* the Tehuantepec fracture zone, *TMVB* the Trans-Mexican volcanic belt, *LTVF* the Los Tuxtlas Volcanic Field, *MCVA* the Modern Chiapanecan volcanic arc, *CAVA* the Central American volcanic arc. The maps are generated using the Generic Mapping Tools (GMT) version 5.3.1 (https://www.generic-mapping-tools.org).

The Middle American subduction system provides an iconic setting to investigate the relation between subduction dynamics and arc magmatism/volcanism. The Rivera microplate and the Cocos plate were formed during the fragmentation of the ancient Farallon plate at ~ 25–10 Ma^1,2^. The subduction of the oceanic Rivera and Cocos plates beneath the North American, Caribbean, and Panama plates has resulted in a ~ 2700-km-long convergent margin (Fig. 1). The Rivera microplate subducts steeply at a dip angle of ~ 50° and a slow rate of ~ 3 cm/yr^2,3^. The Cocos plate demonstrates both normal and flat subduction, and the convergence rate at the trench increases from ~ 5 cm/yr southeastward to ~ 9 cm/yr^4^. The subduction of the Cocos plate beneath the North American-Caribbean plate boundary has formed a subduction‐subduction‐transform triple junction. The rough-smooth boundary, which separates the Cocos lithosphere generated at the East Pacific Rise and the Cocos–Nazca spreading center, is subducted slightly to the south of the Caribbean–Panama plate boundary (Fig. 1). The oceanic crust within the Cocos plate is smooth and has low magnetic anomalies to the north of the rough–smooth boundary, and is characterized by rough seamounts and strong magnetic anomalies to its south^5,6^.

The active volcanoes are extremely unevenly distributed along the Middle American subduction margin (Fig. 1). Specifically, the Trans-Mexican volcanic belt (TMVB) aligns roughly along the 100-km plate interface, except its southeastern end which is further inland and nearly perpendicular to the trench. The Central American volcanic arc (CAVA) is densely spaced and parallel to the trench, which represents the typical subduction-related arc volcanism. The volcanoes are very sparse between the TMVB and the CAVA, and only include the Los Tuxtlas volcanic field and the small, isolated Modern Chiapanecan volcanic arc (MCVA)^7^. Most of the volcanoes along the Middle American subduction margin are dominantly calc-alkaline, demonstrating that the subduction-induced magmatism serves as the primary origin; However, the presence of small volumes of intraplate alkaline lavas indicates contributions of partial melts from other sources, such as the subducting plate, deep mantle, or active continental rifting^8–10^.

In this study we seek correlations between the seismic characteristics of the subducting slab and the overlying mantle wedge and the distribution patterns of volcanism along the Middle American subduction system. A high-resolution image of the lithospheric structure for the entire Middle American subduction system is constructed using an advanced full-wave propagation simulation and inversion method. All the broadband seismic stations deployed from 1997 to 2019 in our study area are included (Fig. 1; Table S1), allowing us to extract useful Rayleigh-wave signals at a broad range of periods (Fig. S1). By cross-correlating ambient noise seismic data between station pairs, the raypath coverage is significantly increased within the entire study area, including the regions with limited coverage of seismic stations and the immediately offshore region (Figs. S2 and S3). The final seismic tomographic model is achieved after three iterations of wave simulation and inversion (Figs. S4–S9).

### Model resolution tests

We conduct a variety of model resolution tests in order to validate the major seismic features to be discussed in this study. To quantitatively evaluate the model recovery tests, we refer to a >  = 70% recovery of the input amplitude as resolvable. Note that we only run one iteration for the resolution tests (instead of three iterations as we do to achieve the final model), which partly explain why the amplitude of the input model cannot be fully recovered at certain regions.

We first perform a series of checkerboard resolution tests for the horizontal dimension (Figs. S10–S13). For the northwestern and southeastern portions of the study region, the shear-wave velocity model can be well recovered at a depth range of ~ 5–120 km, and the minimum resolvable dimensions increase horizontally from 110 to 220 km with increasing depths. For the central portion, the model resolution is lower with a resolvable horizontal dimension of 110–165 km at the depths of 20–65 km and ~ 220 km at greater depths. We then set up the checkerboard resolution tests with the cell dimension varying with depths (Figs. S14 and S15). For an input model with a sharp velocity boundary at 100 km depth, the pattern and amplitude of the input checkerboard can be well recovered at the depths <  = 100 km through the study region (Fig. S14). At depths greater than 100 km, the alternate patterns of the input checkerboard can be much better resolved in the southeastern portion of our study region than in the northwestern portion. For an input model with two sharp velocity boundaries at the depths of 50 km and 150 km, the alternate pattern and amplitude of the input checkerboard can be fairly well recovered at the depths <  = 150 km through the study region (Fig. S15). At depths greater than 150 km, we are only able to resolve the alternate patterns in the southeastern portion. Furthermore, to evaluate the impact of the data uncertainties on the model resolution, we conduct the checkerboard resolution tests with random noise added. We set the noise as the standard deviation of the phase delay measurements after the 3rd iteration. The resolution tests demonstrate that the pattern and amplitude of the input checkerboard can be well recovered with the random noise added (the third column in Figs. S14 and S15).

The checkerboard resolution tests demonstrate some along-strike smearing effect, which is mainly observed offshore, due to the prevalent NW–SE orientation of the raypath (Figs. S2 and S3). We conducted a resolution test to evaluate the impact of the raypath direction on the resolved structures (Fig. S16). We set up an input model with five high-velocity anomalies oriented in the SW-NE direction. The recovered model demonstrates that the SW-NE trending pattern of the five high-velocity anomalies can be well constrained down to ~ 150 km depth. Even though along-strike smearing is observed at greater depths due to the limited raypath coverage, we are able to partially recover the pattern and the amplitude of the five high-velocity anomalies.

We then carry out the model resolution tests for the geometry of the subducting slabs (Figs. S17–S23). We first test a simple model with a − 10% velocity perturbation for the top 40 km and a + 10% velocity perturbation at greater depths (Figs. S17 and S18). Figure S18 shows that the sharp velocity boundary can be well recovered, though the velocity amplitude of the high-velocity lithosphere cannot be fully recovered. A simple test with a vertical high-velocity slab at the depths of 40–200 km beneath the subduction margin shows that the slab can be well recovered in the northwestern and southeastern portions of the study region, and can only be partially recovered in the central portion (Figs. S19 and S20). The model recovery test for slab segmentations along strike supports that we are able to reveal a slab gap at the scale of >  = 200 km (Figs. S21 and S22). We then setup a model with a dipping subducting slab and a flat segment (Fig. S23). The model recovery demonstrates robust constraints on the dipping (and thickness) of the subducting plate in the northwestern and southeastern regions. More than 70% of the velocity amplitude of the subducting plate can be recovered at the depths of 50–200 km. The low-velocity mantle wedge above the high‐velocity slab can be generally recovered. However, the velocity amplitude of the flat slab segment can only be partially recovered at the depths of ~ 50–100 km, but can be better recovered at depths greater than 100 km where the slab replunges into the upper mantle.

We validate the model resolution in the central portion of the Middle American subduction system (Fig. S24). The input model includes two vertical high-velocity anomalies, representing the central Cocos slab and the subducted Yucatan slab, respectively. These two high-velocity anomalies are about 200 km apart. Our resolution test shows that our tomographic model can well resolve the two high-velocity anomalies down to ~ 130 km depth. A low-velocity gap between the two high-velocity anomalies is expected to be well imaged.

In summary, the model resolution tests demonstrate that our tomographic method has the capability to image the three-dimensional geometry of the subducting slab in the Middle American subduction system. Nevertheless, we acknowledge that the velocity amplitude of an input model cannot be fully recovered at certain regions (Figs. S10–S24). The apparent heterogeneity of the seismic velocities of the slab along strike and down dip can be partly due to the uneven raypath coverage (Figs. S2 and S3). Some features in our tomographic model, especially the offshore region and at greater depths, may reflect the along-strike smearing due to the SE-NW directional raypath coverage. Nevertheless, this study focuses on the seismic features at the regions and depths with the best model resolutions, which are tested to be recoverable through our model resolution tests.

### Seismic structure revealed by ambient noise tomography

The subducted oceanic plates are commonly imaged as high-velocity seismic features from the trench dipping landward down to the upper mantle. Our model covers the subduction of the southernmost tip of the Rivera subduction system and the entire Cocos plate along the Middle American subduction margin. We divide the Cocos plate into the northern, central, and southern segments, primarily based on the distribution patterns of the active volcanoes. The northern segment is bounded by the El Gordo Graben and the O'Gorman fracture zone; The central segment is located between the TMVB and the CAVA; and the southern segment corresponds to the along-strike distribution of the CAVA.

#### The Rivera-Cocos transitional zone

**Figure 2:**
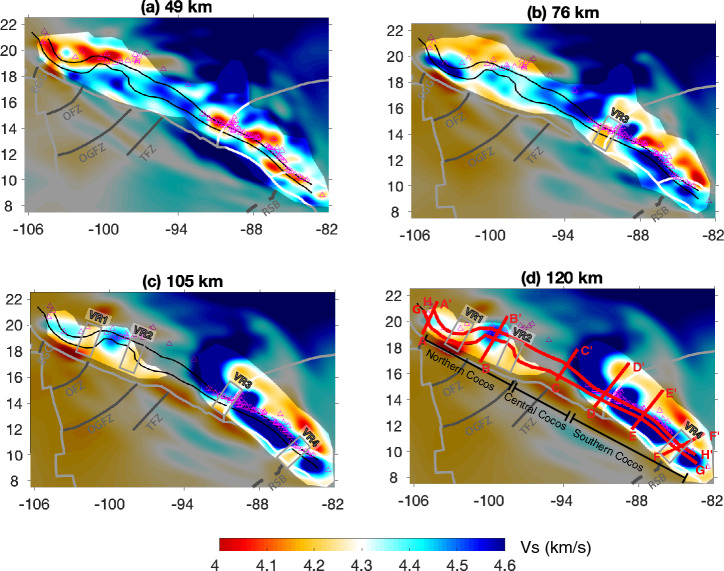
(**a**-**d**) Shear-wave velocities at the depths of 49 km, 76 km, 105 km, and 120 km. The gray shaded areas mask the regions with low resolutions (i.e. less than 70% recovery of the input velocity perturbation) based on checkerboard resolution test in Fig. S12. The distinct velocity reductions are labeled as VR1, VR2, VR3, and VR4. The red lines in (**d**) mark the profile locations in Figs. 3 and 4. Other symbols are the same as in Fig. 1.

**Figure 3:**
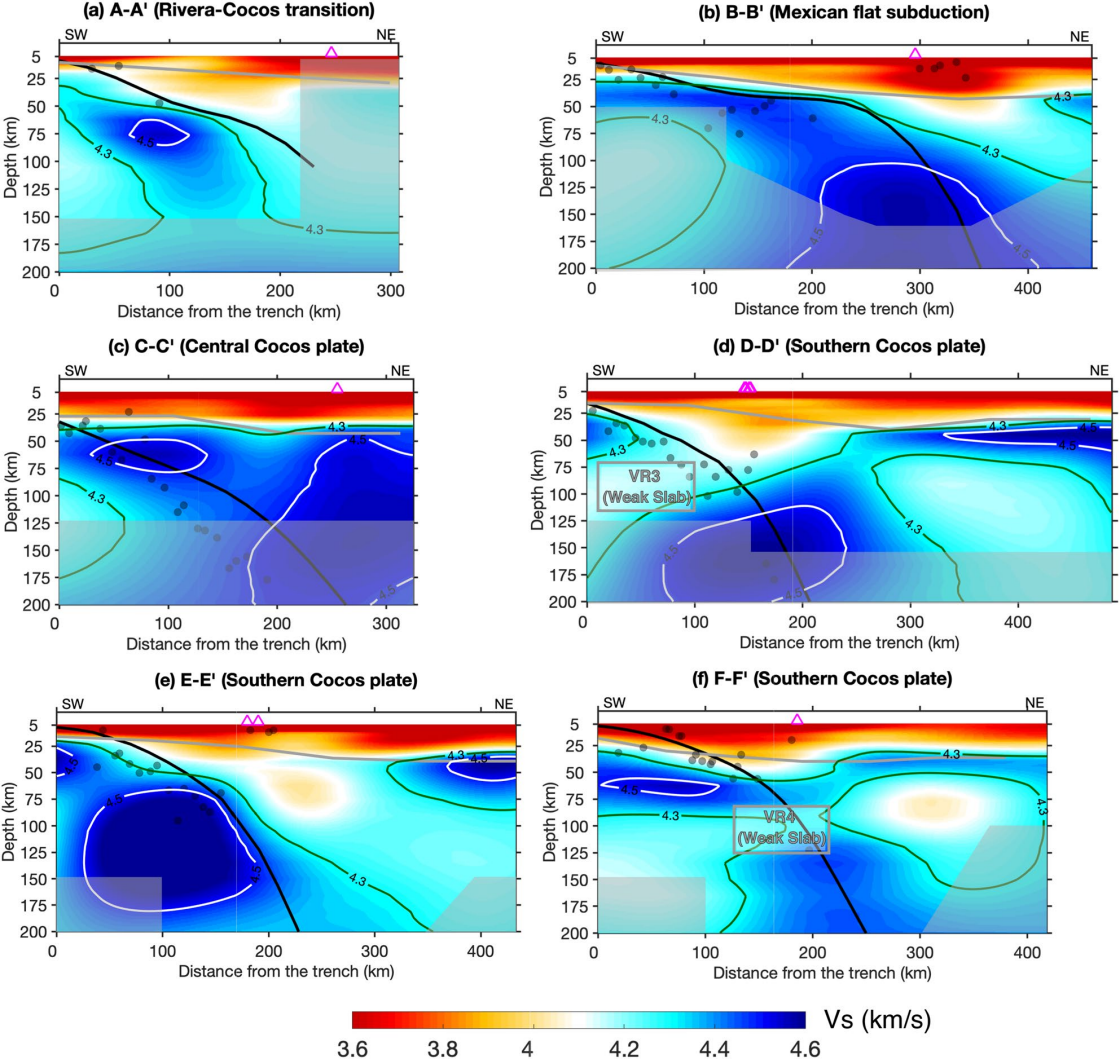
(**a**-**f**) Vertical cross-sections of the tomographic model at the depths of 5–200 km. The thick black lines represent the plate interface of Slab2.0^11^. The gray lines represent the Moho depth extracted from CRUST 1.0^61^. The white lines represent the Vs = 4.5 km/s contours. The gray lines represent the Vs = 4.3 km/s contours. The gray dots are the earthquakes, and the magenta triangles are the surface volcanoes. The gray shaded areas mask regions with low model resolutions (i.e. less than 70% recovery of the input velocity perturbation) based on the model recovery test for the geometry of the subducting slabs in Fig. S18.

Our model images the oceanic lithosphere beneath the El Gordo Graben, which separates the Rivera and Cocos plates, as a continuous feature extending from the trench down to ~ 150 km depth (Figs. 2 and 3a). The Vs of the oceanic lithosphere falls within a range of ~ 4.4–4.7 km/s, with the highest amplitude at the depths of 50–100 km, where we have the best model resolution. The top of the imaged oceanic lithosphere roughly follows the plate interface of the Slab2.0 model^11^. The Vs beneath the oceanic lithosphere is about 4.1–4.3 km/s, slightly lower than the global average of the oceanic asthenosphere (~ 4.3–4.5 km/s^12^). We observe a low-velocity mantle wedge with Vs of ~ 3.8–4.2 km/s (Fig. 3a), which is at least 7% lower than the global average (Vs of 4.5 km/s) of the mantle lithosphere.

#### The northern Cocos

The northern segment of the Cocos slab is characterized by the Mexican flat subduction, which is bounded roughly between the Orozco and O'Gorman fracture zones (Figs. 2, 3b, and S25). The Mexican flat slab extends laterally for a distance of ~ 200 km at shallow depths and then resumes normal subduction into the mantle at greater depths (Fig. 3b), which has been previously imaged by seismic body wave tomography^13^. The slab appears to be thicker and flatter with increasing depth, which likely reflects the limitation of the vertical model resolution as demonstrated by the resolution test (Fig. S23b). The Vs within the mantle wedge, where normal subduction resumes, is on average less than 4.2 km/s. We image a distinct low-velocity (Vs <  = 3.6 km/s) anomaly within the overlying North American continental crust, which roughly correlates with the TMVB and a cluster of shallow seismicity (Fig. 3b). This crustal low-velocity anomaly is at least ~ 5% lower than the average velocity of its surroundings.

**Figure 4:**
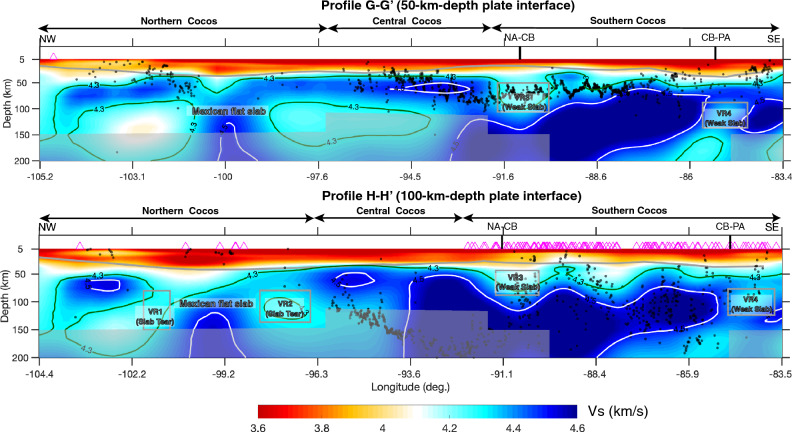
Vertical cross-sections of the tomographic model along the plate interface at the depths of 50 km and 100 km. The vertical black lines mark the North American-Caribbean plate boundary (NA-CB) and the Caribbean-Panama plate boundary (CA-PA). Other symbols are the same as in Fig. 3. The gray shaded areas mask regions with low model resolutions (i.e. less than 70% recovery of the input velocity perturbation) based on the model recovery test for the geometry of the subducting slabs in Fig. S18.

When the subducting slab transits from normal subduction to nearly flat subduction, it can either bend smoothly or tear apart. The northern Cocos slab is imaged as a continuous high-velocity feature after its subduction at the trench, with an average Vs of 4.4–4.6 km/s (Fig. 4a). At greater depths, our model appears to image a narrow flat-normal transitional zone at the northwestern edge of the Mexican flat slab and a broad transition at its southeastern edge. Specifically, to the northwestern edge of the flat slab, we observe a high-velocity slab extending down to about 85 km depth, which is underlain by a subparallel low-velocity anomaly (Fig. S26a). Within the transitional mantle wedge, we observe a low-velocity feature (Vs <  = 4.3 km/s) extending from the backarc tilting upward toward southwest to beneath the volcanoes (Fig. S26a). In comparison, to the southeastern edge of the flat slab, the slab subducts from the trench down to about 50 km and then becomes nearly flat for a lateral distance of a couple of hundred kilometers (Fig. S26b). Such a shallowly-dipping slab was also previously imaged by the teleseismic receiver function analysis^14^. Our tomographic model shows significant velocity reductions (Vs <  = 4.3 km/s) at both edges of the Mexican flat subduction (VR1 and VR2 in Figs. 2c, d, and 4b), which likely indicate the presence of slab tearing.

#### The central Cocos

The central segment of the Cocos plate dips northeastward at a shallow angle, with an average Vs of 4.4–4.6 km/s (Figs. 2, 3c, and 4). We don’t observe a typical low-velocity mantle wedge within this portion of the subduction system. Instead, a large-scale high-velocity (Vs of ~ 4.5–5.0 km/s) feature is revealed down to the bottom of our model beneath the North American plate (Figs. 2 and 3c), which has also been observed by the global-scale seismic model^15^ and the receiver function analysis^16^. Interestingly, both the Los Tuxtlas volcanic field and the MCVA are located at the margins of slow-fast velocity transitions at all the depths (Fig. 2). For example, the Los Tuxtlas volcanic field is located at the southeastern tip of a low-velocity backarc (Figs. 2a and b), and the MCVA is located at the western margin of the large-scale high-velocity feature (Figs. 2b–d). To the southwest of the MCVA, the seismic velocities in the crust and uppermost mantle wedge appear to be slightly lower than its surroundings (Fig. 2a and b).

#### The southern Cocos

In general, the southern Cocos plate appears to be thicker and seismically faster compared to the northern and central segments (Figs. 2, 3, 4). The southern Cocos plate is characterized by strong velocity variations in both along-strike and downdip directions (Figs. 2b–d, 3d–f, 4 and S25). First, we observe clear along-strike velocity reductions of the southern Cocos plate near the North American-Caribbean plate boundary (VR3) and the Caribbean-Panama plate boundary (VR4; Figs. 2b–d). For example, at 100 km depth, the average Vs of the southern Cocos plate is ~ 4.8 km/s, with exceptional low Vs values of ~ 4.0–4.2 km/s near the two plate boundaries (Fig. 2c). Interestingly, the strongest velocity reduction is observed at shallower depths of 50–100 km near the North American-Caribbean plate boundary and at greater depths of 85–125 km near the Caribbean-Panama plate boundary (Figs. 2b–d, 3d, f, and 4). Second, the shear-wave velocity along the downdip direction of the subducting slab varies within a broad range; For example, along profile D-D′, the slab signature is absent at depths shallower than 100 km and is clearly imaged at greater depths (VR3 in Fig. 3d). Along profile E-E′, the Vs is less than 4.5 km/s near the trench at the depths of ~ 50–75 km and has the strongest amplitude at depths greater than 100 km (Fig. 3e). Along profile F-F′, the slab is imaged to be nearly flat at shallow depths and then subducts steeply at greater depths, and the seismic velocity is reduced near the transitional depths (~ 75–100 km) (VR4 in Fig. 3f). In addition, a distinct low-velocity anomaly is observed beneath the subducting plate at the depths of ~ 125–175 km (Fig. 3f). A low-velocity mantle wedge is well imaged above the southern Cocos slab, with the Vs varying at ~ 3.7–4.2 km/s (Figs. 2, 3d–f, and 4). Along profile E-E’, we observe a typical low-velocity mantle wedge with Vs < 4.0 km/s, extending from ~ 100 km depth upward to beneath the volcanic arc (Fig. 3e). Along profile D-D’, the low shear-wave velocities extensively distribute from the oceanic asthenosphere through the subducting slab to the mantle wedge directly beneath arc volcanoes, following the velocity contour of 4.3 km/s (Fig. 3d). We observe a low-velocity column extending from the oceanic asthenosphere penetrating through the subducting slab to the mantle wedge directly beneath the arc volcanoes (Fig. 3d). Along profile F-F′, a low-velocity anomaly is located to the southwest of the volcanic arc, which appears to have no deep source (Fig. 3f). In addition, we observe a thin high-velocity (Vs of ~ 4.5 km/s) layer beneath the continental crust, which we interpret as the continental lithosphere of the Caribbean plate and the Panama microplate (Fig. 3d–f).

## Discussion

Our new model provides a comprehensive map of the entire Middle American subduction system at a depth range of 15–180 km, extending from the trench to the backarc. Our model reveals that the subducting Cocos slab varies significantly along strike and down dip in terms of geometry and seismic velocity (Figs. 2, 3, 4). Further, our model demonstrates the seismic differences of the mantle wedge along the entire subduction margin, providing new seismic constraints on the unusual distribution patterns of arc volcanism. Even though many seismic studies have been carried out in our study region^17–20^, most existing models only cover a portion of the Middle American subduction system. A recently constructed seismic anisotropy model has revealed the large-scale flow patterns within the mantle of the Middle American and Caribbean subduction systems^21^, but is lack of resolution for the detailed slab geometry and mantle wedge at the depths shallower than 200 km. Below we first discuss the implications of the imaged velocity reductions within the slab and then explore the relation of the subduction dynamics with the arc magmatism/volcanism.

First, is the subducting Cocos slab weakened or broken along strike and down dip? We propose that the velocity reductions within the normal-subduction slab near the trench indicate a weak segment of the slab (Figs. 3a, d–f). The continuity of the subducting slab is further supported by the existence of seismicity along the plate interface (Figs. 1 and 3) and the detection of a typical corner flow within the mantle wedge of the southern Cocos plate^21^. Here we consider two primary factors that can significantly reduce the seismic velocity of the subducting slab near the trench. The first factor is bending of the oceanic plate near the trench, which can lower the strength of the slab and reactivate the existing faults/fractures and/or form new bending-related faultings^22,23^. In this scenario, sea water can penetrate downward into the oceanic crust (and even into the oceanic mantle lithosphere), which would significantly hydrate and alter the subducting slab and thus reduce the seismic velocities^24^. The presence of low velocities within the southern Cocos slab near the trench was previously imaged and interpreted as the hydrated oceanic crust based on petrological modelling^25^, supporting our hypothesis here.

The second contribution to the reduction of seismic velocities within the slab is the underlying oceanic asthenosphere. The low-velocity feature observed beneath the subducting plate (Figs. 3 and S26a) has also been imaged at other subduction zones^26–29^. The low-velocity layer at the bottom of the oceanic lithosphere could indicate the presence of partial melts, fluids, shearing, and/or volatiles^25–31^. Regardless of the disagreement on the nature of the low velocities imaged beneath the slabs, many studies suggest that the presence of this low-velocity anomaly can have a potential impact on the overlying slab over geologic time. The numerical modeling reveals low viscosity and high strain rate within and beneath the subducting slab near the trench^33^, supporting the reduction of the slab strength after subduction. Our tomographic model suggests that the subducting slab is likely weakened by the underlying low-viscosity oceanic asthenosphere, especially near the North American-Caribbean plate boundary and the Caribbean-Panama plate boundary (Figs. 3d and f). The kinematic model demonstrates that the transform motion between the North American and Caribbean plates doesn’t have a considerable impact on the deformation of the subducting plate^34^. In addition to the above factors, the differences in the age and properties of the Cocos oceanic lithosphere across the rough-smooth boundary could have contributed to the velocity reduction (VR4) observed near the Caribbean-Panama plate boundary. For example, it has been suggested that the subducting Cocos slab could be contorted across the rough-smooth boundary^35^, which would consequently reduce the strength of the slab. Alternatively, the low seismic velocity within the subducting slab near the Caribbean-Panama plate boundary has been interpreted as subducted oceanic seamounts or overthickened oceanic crust^17,25^.

Whether a slab tearing exists at the flat-normal transitional subduction has been the subject of many studies at global subduction zones. Our model appears to support the possibility that the slab is tore at both edges of the Mexican flat slab subduction (Fig. 4). The shear-wave velocities where we interpret as slab tearing (Vs <  = 4.3 km/s; VR1 and VR2 in Figs. 2 and 4) are about 4–5% lower than the average Vs of the upper mantle, consistent with the values where slab tearing has been proposed in South America^25,27^. Many studies have suggested that the Cocos plate has been continuously fragmenting near the Orozco fracture zone, the northwestern edge of the Mexican flat slab^4,36,37^. The seismic anisotropy studies observe a toroidal mantle flow near the Orozco fracture zone, supporting slab tearing at the northwestern edge of the flat slab^18^. The southeastern edge of the Mexican flat slab is likely torn along the O'Gorman fracture zone (Fig. 4), which has also been previously proposed^18,38^. Some studies have indicated the fragmentation between the Rivera and Cocos plates^37,39,40^, which is beyond our study region. Nevertheless, not all the studies agree with the hypothesis of slab tearing at the flat-normal transitional subduction regions. For example, the distribution patterns of seismicity, nonvolcanic tremor, and slow slip favor a sharp slab contortion rather than slab tearing at the southeastern edge of the flat slab^41^.

Second, what is the relation of the subducting slabs with the arc magmatism/volcanism? We suggest that the significant three-dimensional variations of the subducting Cocos slab have strongly modified the mantle flow patterns, resulting in the unusual distribution patterns of the volcanism. Our imaging of normal and flat subduction can explain the dominant calc-alkaline magmas observed at the arc volcanoes, which are directly related to the slab dehydration process^42^. Specifically, the normal subduction of the northernmost and southern portions of the Cocos plate has produced a typical low-velocity mantle wedge, in correlation with the northwestern segment of the TMVB and the CAVA, respectively (Figs. 3a, e, and 4). The southeastern TMVB corresponds with the leading edge of the Mexican flab slab subduction and a large crustal low-velocity anomaly (Figs. 3b and 4). This crustal low-velocity anomaly has also been previously detected by the receiver function analysis^43^ and the magnetotelluric study^44^. We propose that the crustal low-velocity anomaly likely indicates the accumulation of magma with partial melts produced by the resumed normal subduction, which can explain the further inland position of the southeastern TMVB volcanism.

The subduction of the central Cocos plate beneath North America doesn’t result in the presence of typical arc volcanoes (Fig. 1). The shallowly dipping slab of the central Cocos with the absence of a typical low-velocity mantle wedge (Figs. 3c and 4) indicates the lack of partial melting in the wedge, which would consequently prevent formation of arc magmatism/volcanism. In addition, the large-scale high-velocity structure imaged beneath the North American plate (Figs. 2 and 3c) is expected to have played a critical role in modifying the subduction dynamics and the mantle flow pattern. A previous study^16^ interprets this high-velocity feature as the subducted southwest-dipping Yucatán slab and suggests that the Yucatán slab has truncated the subducting Cocos slab and resulted in the cessation of arc volcanism at both subduction systems.

Nevertheless, the formation of the magmatism/volcanism cannot be simply explained by the subduction of the Cocos slab. In addition to the typical calc-alkaline magmatic rocks, a small amount of intraplate-type alkaline basalts has been observed at the TMVB volcanoes^45^; the Los Tuxtlas volcanic field contains both alkaline and sub-alkaline magma^8^; and the MCVA has a transitional composition from calc-alkaline to adakitic^46,47^. The diversity of the geochemical signatures reflects a mixture of partial melts resulting from different sources in the mantle. Many hypotheses have been proposed to explain the observed geochemical signatures at those volcanoes^9,19,39,48–53^. For example, many studies propose that slab tearing, which may exist between the Rivera and Cocos plates and at both edges of the Mexican flat slab, can induce asthenospheric upwelling and partial melting^19,39,52^. Some studies suggest that the mantle wedge of the Mexican flat slab is highly heterogeneous and enriched with partial melts, resulting in the alkaline magma^9,48^. The active tectonic extension and continental rifting can also contribute to the unique geochemical signatures^49–51^. A geodynamic modeling study^53^ suggests that a highly serpentinized old fracture zone is located beneath the MCVA, which has induced a deep and strong dehydration process and can explain the unusual magmatism.

Our study alone cannot exclusively identify all the possible sources of partial melts for the volcanism at the Middle American subduction system. Nevertheless, our model observes clear slab tearing at both edges of the Mexican flat slab (Fig. 4), providing seismic evidence to support the importance of slab tearing on the unusual magmatism/volcanism. Furthermore, our model shows that the MCVA is located to the westernmost edge of the high-velocity feature (Figs. 2b–d), which can initiate asthenospheric upwelling and contribute to the formation of the MCVA.

## Conclusions

Our new tomographic model of the entire Middle American subduction system reveals significant three-dimensional variations of the subducting slabs and the mantle wedge. The seismic velocity of the oceanic lithosphere is significantly reduced near the trench after subduction, indicating that the slab is weakened due to bending and by the underlying oceanic asthenosphere. The distinct velocity reductions at the transitional regions from normal subduction to flat subduction support the presence of slab tearing. As expected, the normal subduction segment of the Cocos plate has resulted in a low-velocity mantle wedge and the arc volcanoes. The absence of a typical low-velocity mantle wedge (and thus partial melts) in the central Cocos provides an explanation for the lack of arc magmatism/volcanism in this region. The presence of slab tearing at the edges of the Mexican flat slab has been modifying the mantle flows and contributing to the geochemical signatures in magmatism/volcanism along the Middle American subduction margin.

## Methods

The Rayleigh‐wave empirical Green's functions (EGFs) are extracted from the cross-correlation of the ambient noise data between each station pair. The ambient noise data were recorded from 1997 to 2019 for a total of 402 broadband seismic stations from 8 temporary and 14 permanent networks in our study area (Fig. 1; Table S1). The data processing procedure includes elimination of the instrument response, cutting of the seismic data into a daily length, resampling of the data into a sample rate of 2 points per second, normalization of the waveform with the frequency-time normalization method^54^, and removal of the segments with large earthquakes (M ≥ 5.5). We cross-correlate the daily vertical-to-vertical components of the ambient noise data between each station pair and stack the daily cross-correlations to increase the signal-to-noise ratio. The EGFs are defined as the negative time derivatives of the stacked cross-correlations^55,56^. The EGFs are split into positive and negative time sides. We require the signal-to-noise ratio of the EGFs to be greater than 3 and the inter-station distance at least 0.75-wavelength. We are able to extract useful Rayleigh-wave signals at a broad range of periods of 7–250 s (Fig. S1).

We simulate wave propagation within the 3-D spherical Earth model using the nonstaggered-grid, finite-difference method^57^. Each seismic station serves as a virtual source, and the source is defined as a Gaussian function with a half width of 3 s. The initial reference model composes of a 2° × 2° global velocity model^58^ for the top 396 km and the 1-D AK135 velocity model at greater depths. The model configuration ranges from longitude 81°W to 107°W and from latitude 7°N to 23°N. The depth range of the model is from the surface down to 988 km with the vertical grid increasing from ~ 1.5 km near the surface to ~ 8.8 km at the bottom.

The phase delays are measured by cross-correlating the EGFs and synthetic waveforms at both positive and negative time sides. We first convolve the EGFs with the source time function to account for the finite-frequency nature. We then perform the cross-correlations at nine overlapping period bands, ranging from 7–15 s, 10–25 s, 15–35 s, 25–50 s, 35–75 s, 50–100 s, 75–150 s, to 150–250 s. When comparing the observed and synthetic waveforms, the cross-correlation coefficient is required to be at least 0.50. The phase delay times are determined by taking the average of the measurements at the positive and negative time sides. As shown in Figs. S2 and S3, the raypath coverage of the phase delay measurements varies at different periods. At periods shorter than 25 s, the raypaths between station pairs only cover the northwestern and southeastern parts of the study area. At periods of 25–100 s, the raypath coverage is great for the entire subduction margin from the trench landward to the backarc. At the periods greater than 100 s, the raypath coverage remains dense to the south of 16°N, but is relatively sparse to the north of latitude 16°N. Note that a majority of the raypaths are oriented in the NW–SE direction due to the distribution of the seismic stations (Figs. S2 and S3).

We calculate the three-dimensional finite-frequency sensitivity kernels of Rayleigh waves at the above period bands. The full-wave tomographic method used in this study considers the influence of both Vp and Vs on the propagation of Rayleigh waves^57,59^. The incorporation of Vp in kernel calculation and inversion provides additional constraints for the shallow crust, which significantly reduces the influence of Vp uncertainties on deeper structures. We represent the phase delay time, Δt, as a function of the Vp and Vs perturbations, ${\text{m}}_{{\text{a}}}$ and ${\text{m}}_{{\upbeta }}$ ^55^:

1 $${\Delta t} = \smallint \left[ {{\text{K}}_{{\upalpha }} \left( {{\text{m}}_{0} ,{\text{x}}} \right){\Delta m}_{{\text{a}}} + {\text{K}}_{{\upbeta }} \left( {{\text{m}}_{0} ,{\text{x}}} \right){\Delta m}_{{\upbeta }} } \right]{\text{dV}}$$

where ${\text{m}}_{0}$ is the velocity model used in wave propagation simulation. ${\text{K}}_{{\upalpha }}$ and ${\text{K}}_{{\upbeta }}$ are the Rayleigh-wave sensitivity kernels to Vp and Vs. dV is the volume increment for the integral. We invert the shear velocity perturbations based on a damped least squares scheme^55,56,60^. The damping and smoothing parameters are selected in terms of the tradeoff between the data misfit and the model variance reduction (Fig. S4). The seismic velocity model is progressively updated for a total of three iterations by iteratively minimizing the phase delays (Figs. S5–S9).

### Supplementary Information

Supplementary Information.

## Data Availability

All the continuous seismic data used in this study were requested via the Data Management Center (https://ds.iris.edu) of the Incorporated Research Institutions for Seismology (IRIS). The computer codes for full-wave ambient noise tomography were developed by Dr. Yang Shen at the University of Rhode Island (https://sites.google.com/view/seismo). The codes are available at Github open-access repository (https://doi.org/10.5281/zenodo.4021348). The velocity models generated by this study are provided as Figs. 2, 3, 4 and will be available upon publication of this work.
