# Supplementary material for: Role of subduction dynamics on the unevenly distributed volcanism at the Middle American subduction system
Source: Sci Rep. 2023 Sep 7;13:14697. doi: 10.1038/s41598-023-41740-y (PMC10484906; doi:10.1038/s41598-023-41740-y)
Supplement: Supplementary file 1 — Supplementary Information. [file 41598_2023_41740_MOESM1_ESM.pdf]

Supplementary Information

Role of subduction dynamics on the unevenly distributed volcanism at the Middle American subduction system

Meng Liu* **and Haiying Gao**

Department of Earth, Geographic, and Climate Sciences, University of Massachusetts Amherst, 627 North Pleasant St., Amherst, Massachusetts 01003, USA

*Corresponding author: Meng Liu (mengliu@umass.edu)

Table S1. Summary of the seismic networks used in this study, including the network name, the network code, the deployment period, the number of stations, and the type of channels.

Network	Code	Deployment Period	Number of stations	Channel
Telica Seismic and Deformation Network	6D	2009-2016	7	BHZ/HHZ
Caribbean USGS Network	CU	2006-present	1	BHZ
GEOSCOPE	G	1982-present	2	BHZ
GEOFON	GE	1993-presnet	1	BHZ
Red Sismologica Nacional	GI	1976-present	7	HHZ
Global Seismograph Network - IRIS/IDA	II	1986-prsent	1	BHZ
Global Seismograph Network - IRIS/USGS	IU	1988-presnet	1	BHZ
Seismic Network of the NorthEastern Mexico	MG	2003-present	1	HHZ
Mexican National Seismic Network	MX	2000-present	3	BHZ
Nicaraguan Seismic Network	NU	1975-present	11	BHZ
Observatorio Vulcanológico y Sismológico de Costa Rica	OV	1984-present	54	HHZ
ChiriNet	PA	2000-present	1	HHZ
Servicio Nacional de Estudios Territoriales	SV	2004-present	10	HHZ
Red Sismológica Naciona	TC	2011-present	11	HHZ
Tectonic Observatory	TO	2004-present	136	HHZ
Mapping the Rivera Subduction Zone	XF	2006-2007	50	BHZ
Imaging the Seismogenic Zone with Geodesy and Seismology	XY	1999-2001	11	BHZ
Broadband Tomography Under Costa Rica and Nicaragua	YO	2003-2006	48	BHZ
Nicoya Seismogenic Zone	YZ	2009-2014	16	BHZ
The Colima Deep Seismic Experiment: Imaging the Magmatic Root of Colima Volcano	ZA	2006-2008	22	HHZ
Rapid Aftershock Deployment for the September 2017 M=8.1 and M.7.1 Earthquakes in Mexico	ZB	2017-2018	6	HHZ
Costa Rica Poco Sol	ZX	2005-2007	2	BHZ

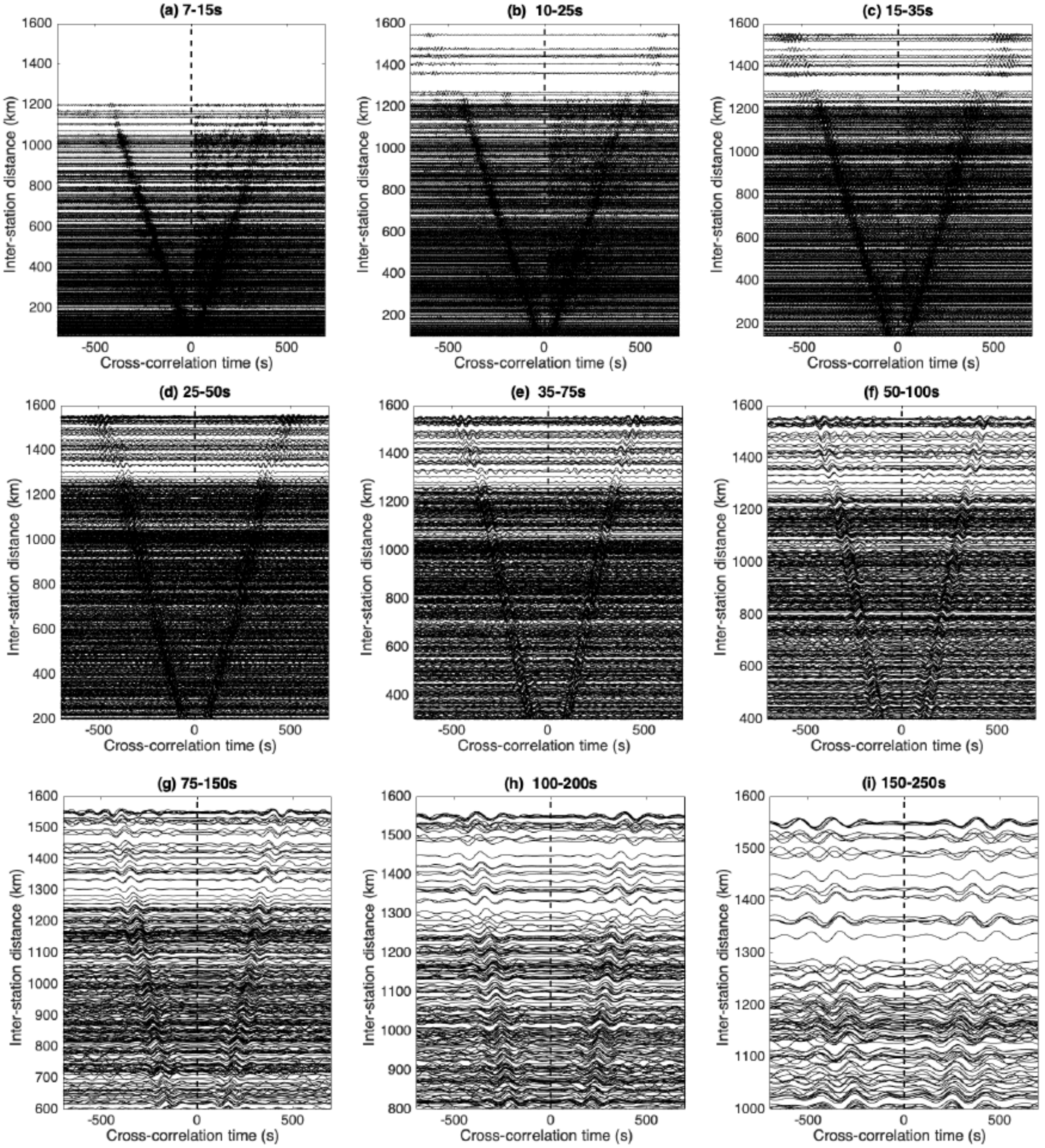

Figure S1. Examples of empirical Green's functions between station pairs, filtered at periods of (a) 7-15 s, (b) 10-25 s, (c) 15-35 s, (d) 25-50 s, (e) 35-75 s, (f) 50-100 s, (g) 75- 150 s, (h) 100-200 s, and (i) 150-250 s.

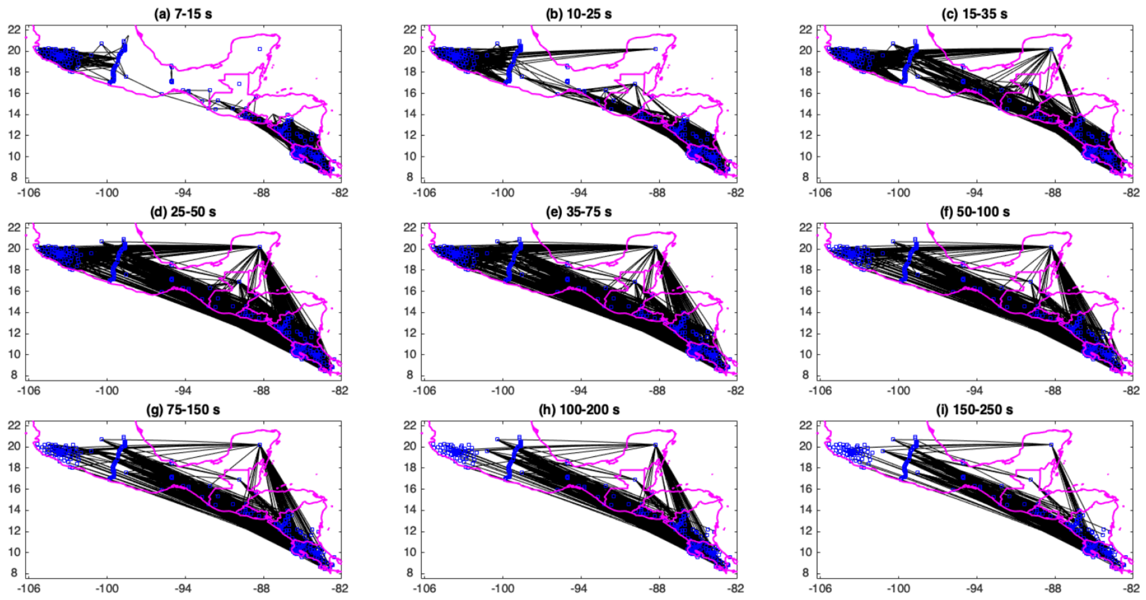

Figure S2. Raypath coverage map for all station pairs at the nine period bands. The blue squares represent the seismic stations. The black lines represent the raypaths between the station pairs.

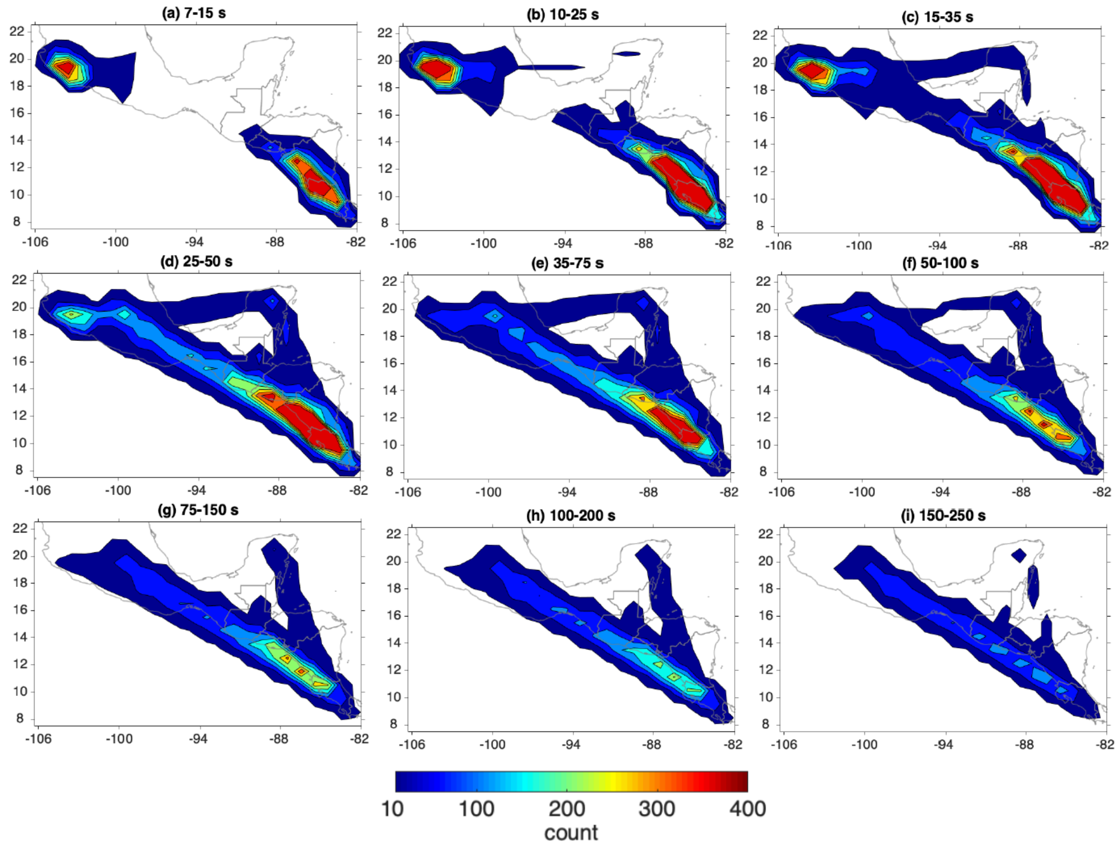

Figure S3. Raypath contour map at the nine period bands. The coverage is color-coded by the number of seismic rays within each 1° × 1° grid.

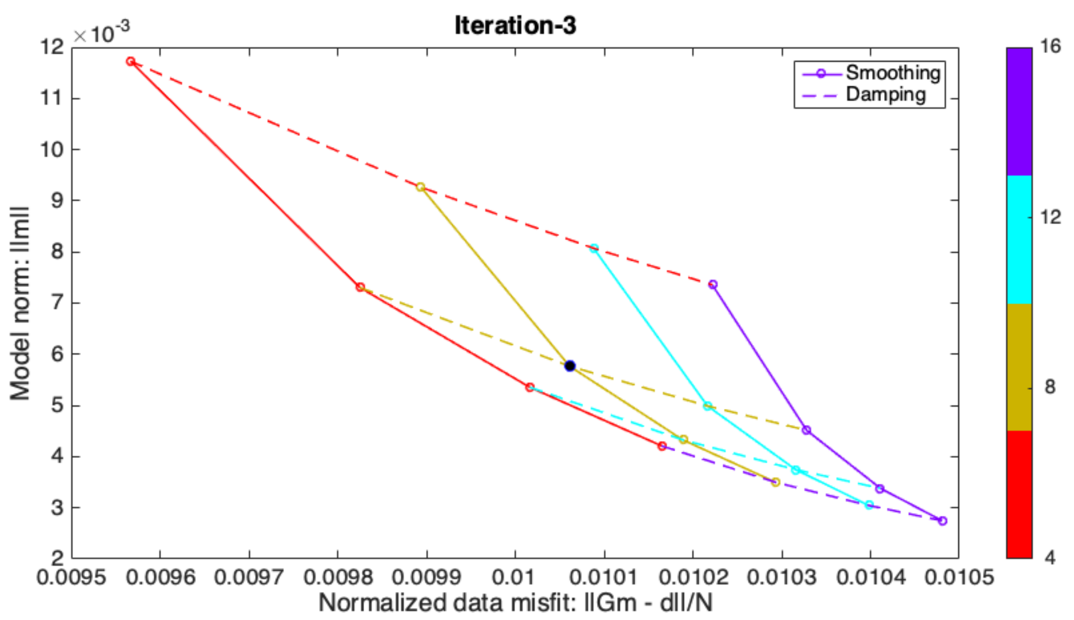

Figure S4. Model norm and normalized data misfit with different smoothing and damping parameters for the final iteration. The color-coded curves represent the values of damping (dashed lines) and smoothing (solid lines) parameters. The blue dot denotes the parameters used for tomographic inversion (smoothing = 8; damping = 8).

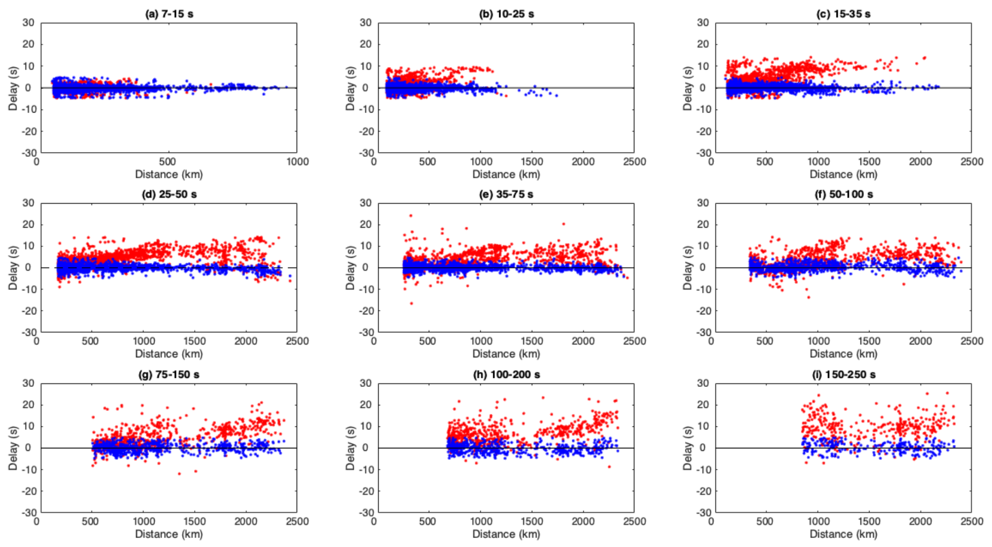

Figure S5. Distribution of phase delay measurements between observed and synthetic waveforms versus inter-station distance at nine period bands. The red and blue dots are phase delays measured from the initial reference model and our final tomographic model, respectively.

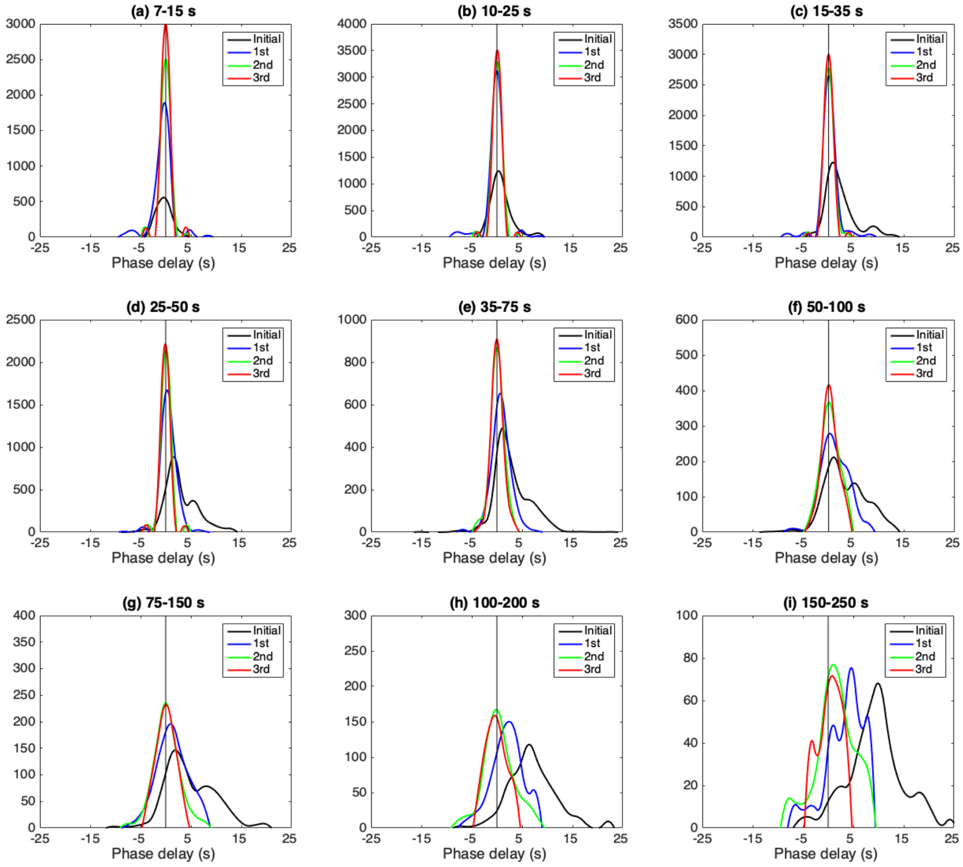

Figure S6. Distribution of phase delays between observed and synthetic waveforms at nine period bands from each iteration. The curves are color-coded by different iterations.

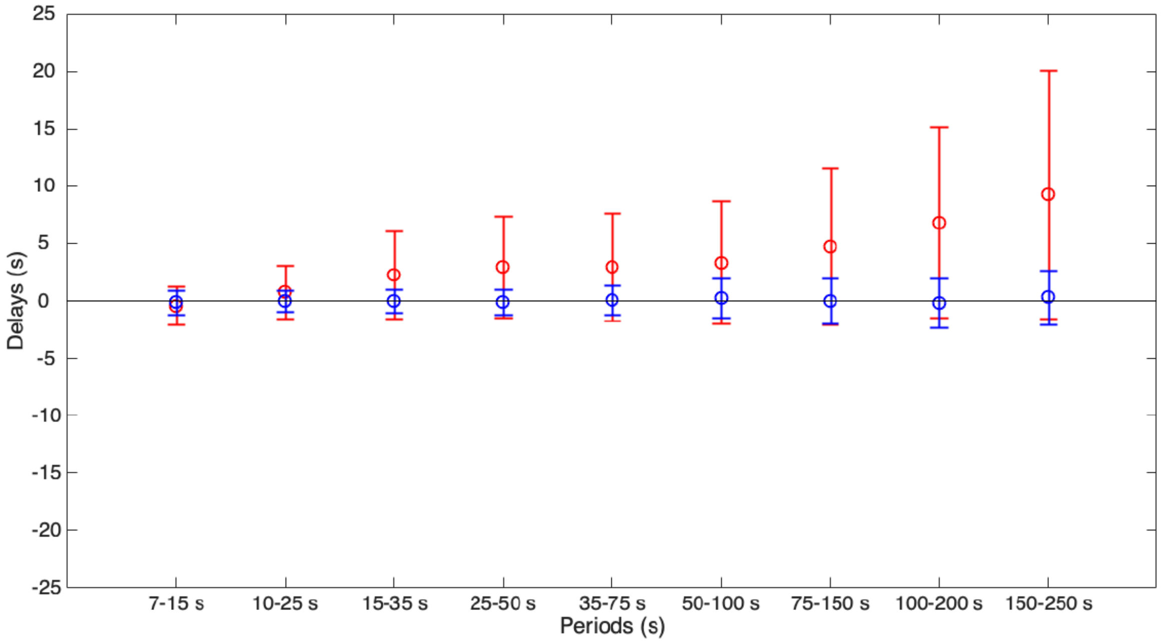

Figure S7. The average phase delays between the initial reference model (red) and our final tomographic model after three iterations (blue) at nine period bands. The error bars represent the standard deviations at each period band.

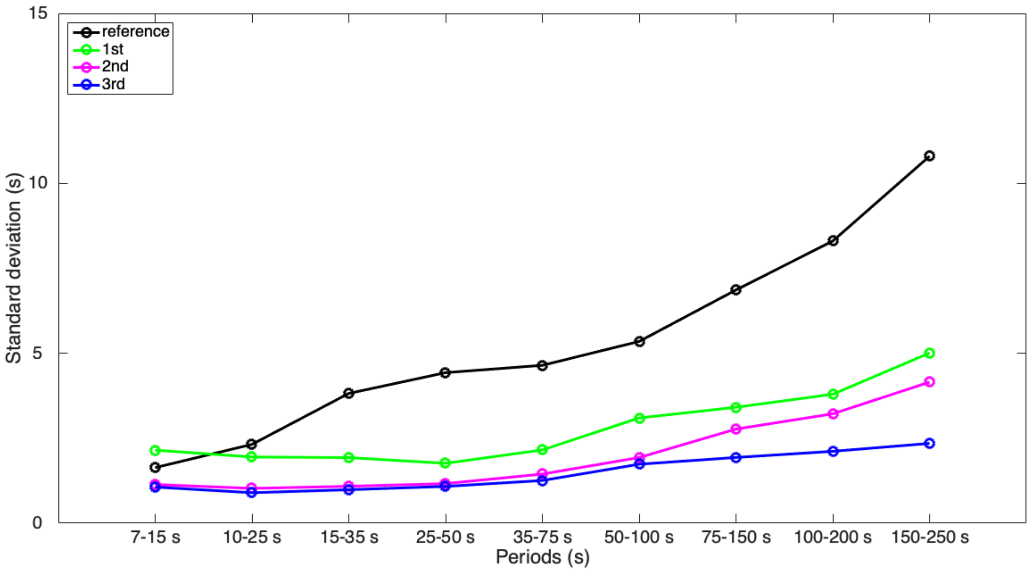

Figure S8. The standard deviations of phase delays for each iteration at nine period bands.

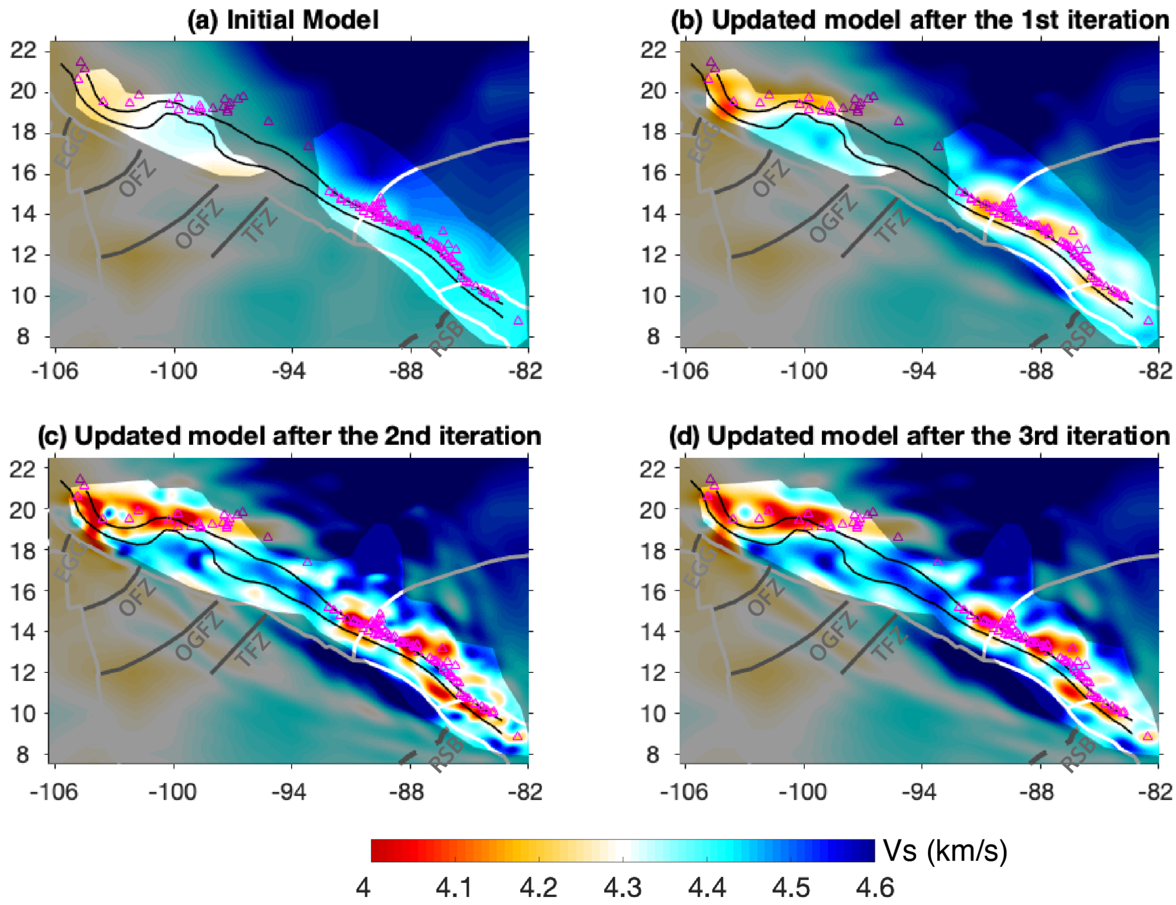

Figure S9. Comparison of the shear-wave velocity models at the depth of 49 km for the three iterations. All symbols are the same as in Fig. 2. The gray shaded areas mask the regions with low resolutions (i.e. less than 70% recovery of the velocity perturbation) based on the checkerboard resolution test in Fig. S12.

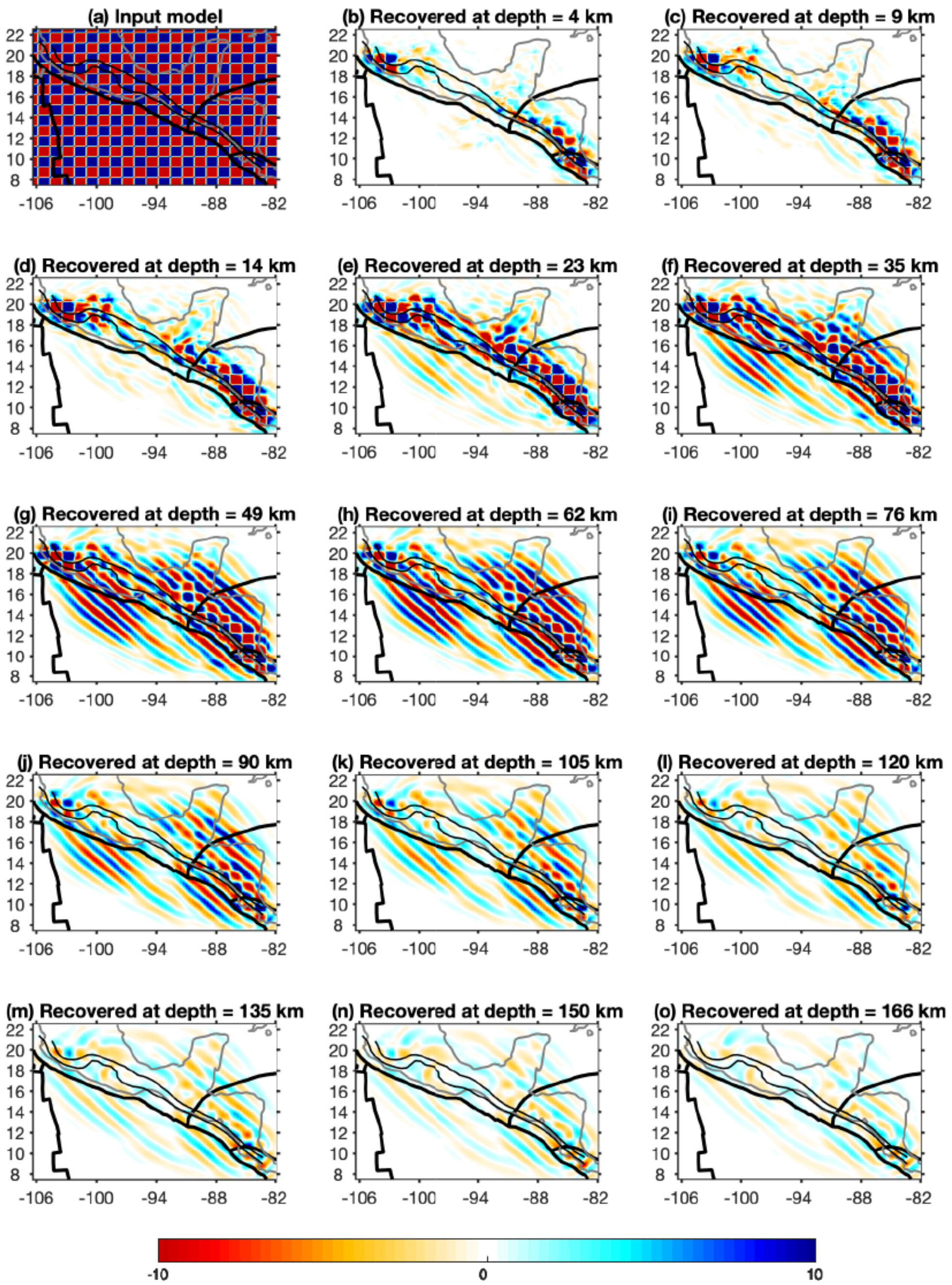

Figure S10. The horizontal checkerboard resolution tests at depths of 0-200 km. (a) The horizontal dimension of the cells is 110 km. The velocity perturbation varies within a range of ±10%. (b)-(o) are the recovered results at multiple depths. The thick black lines mark the major plate boundaries. The gray lines mark the coastline. The black contours represent the plate interfaces at the depths of 50 km and 100 km extracted from Slab2.0[1].

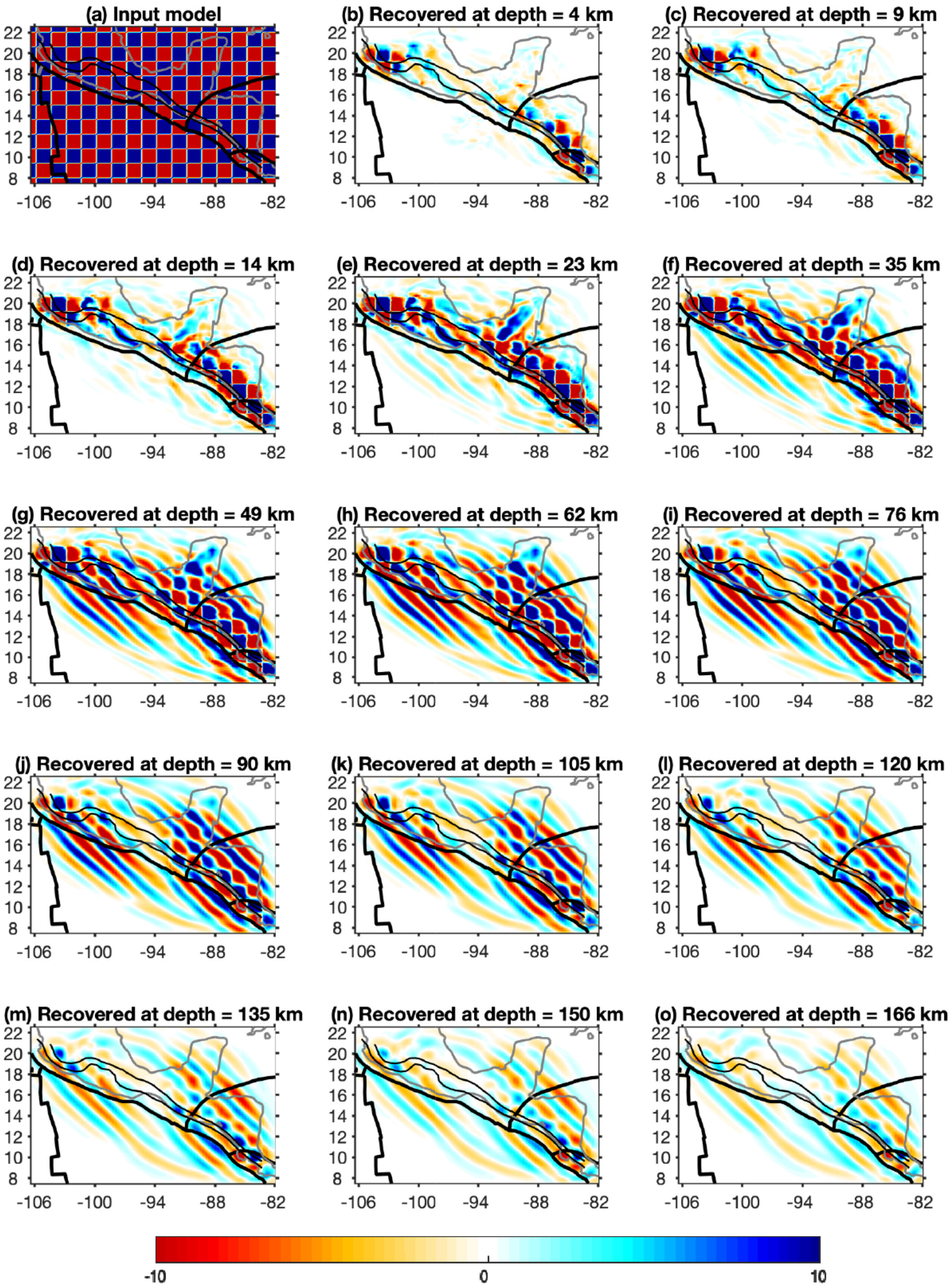

Figure S11. The horizontal checkerboard resolution tests at depths of 0-200 km. (a) The horizontal dimension of the cells is 165 km. The velocity perturbation varies within a range of ±10%. (b)-(o) are the recovered results at multiple depths. All symbols are the same as in Fig. S10.

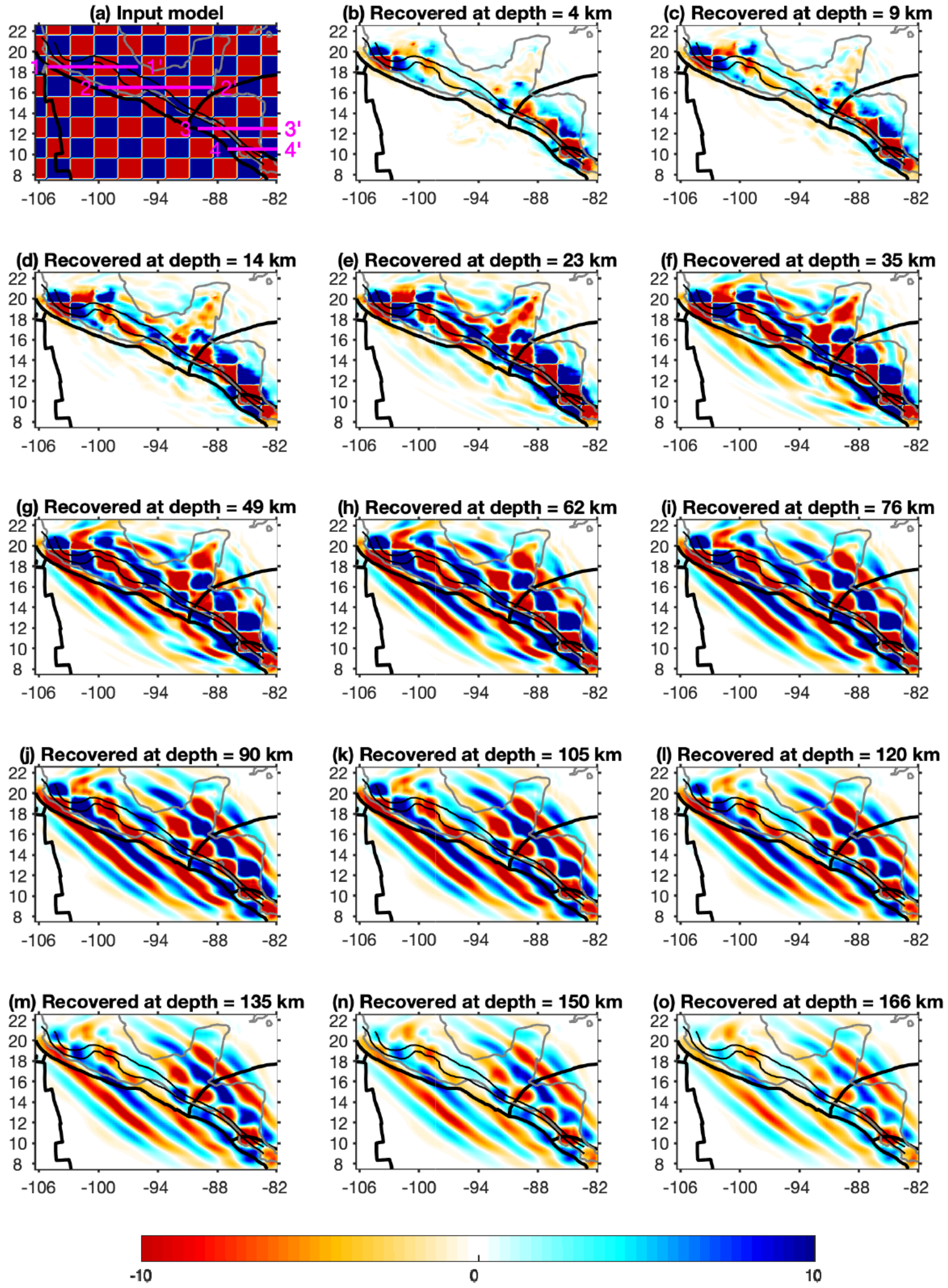

Figure S12. The horizontal checkerboard resolution tests at depths of 0-200 km. (a) The horizontal dimension of the cells is 220 km. The velocity perturbation varies within a range of ±10%. (b)-(o) are the recovered results at multiple depths. The magenta lines show the profiles locations in Fig. S13. Other symbols are the same as in Fig. S10.

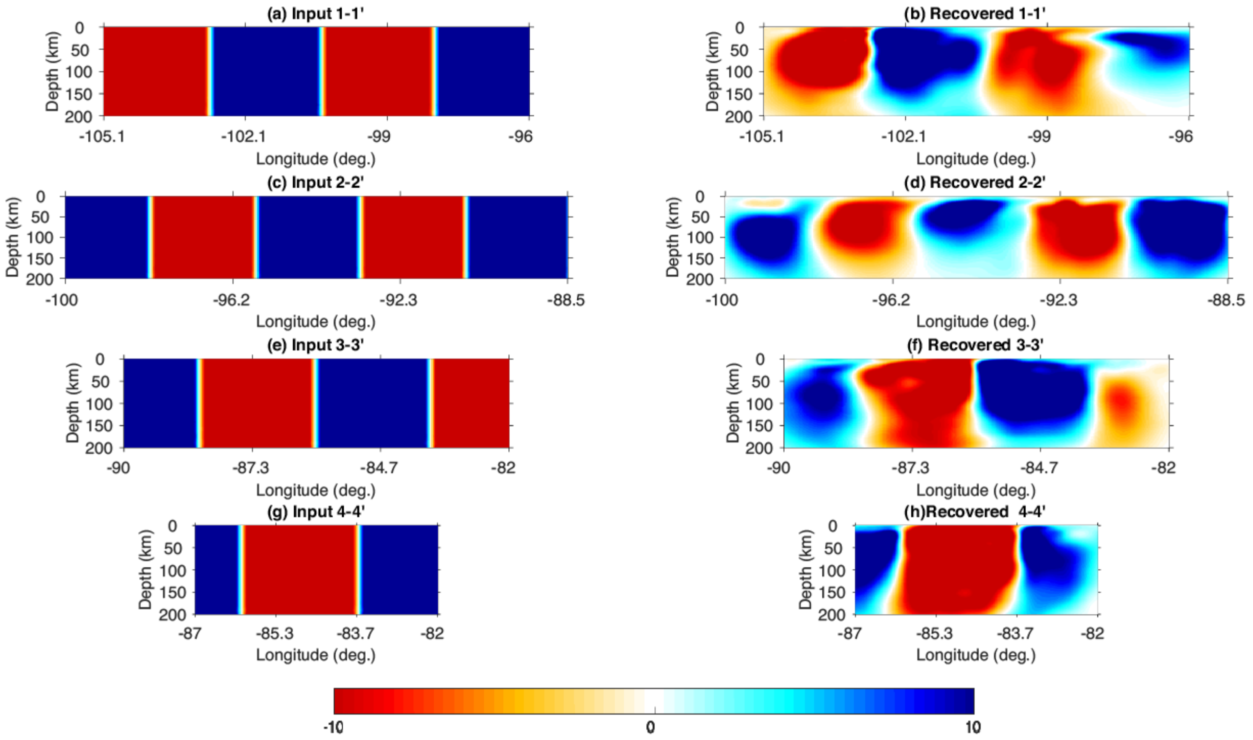

Figure S13. The vertical checkerboard resolution at depths of 0-200 km. The velocity perturbation varies within a range of ±10%. The left column shows the vertical profiles for the input model. The recovered models are displayed to the right, correspondingly. See the profile locations in Fig. S12.

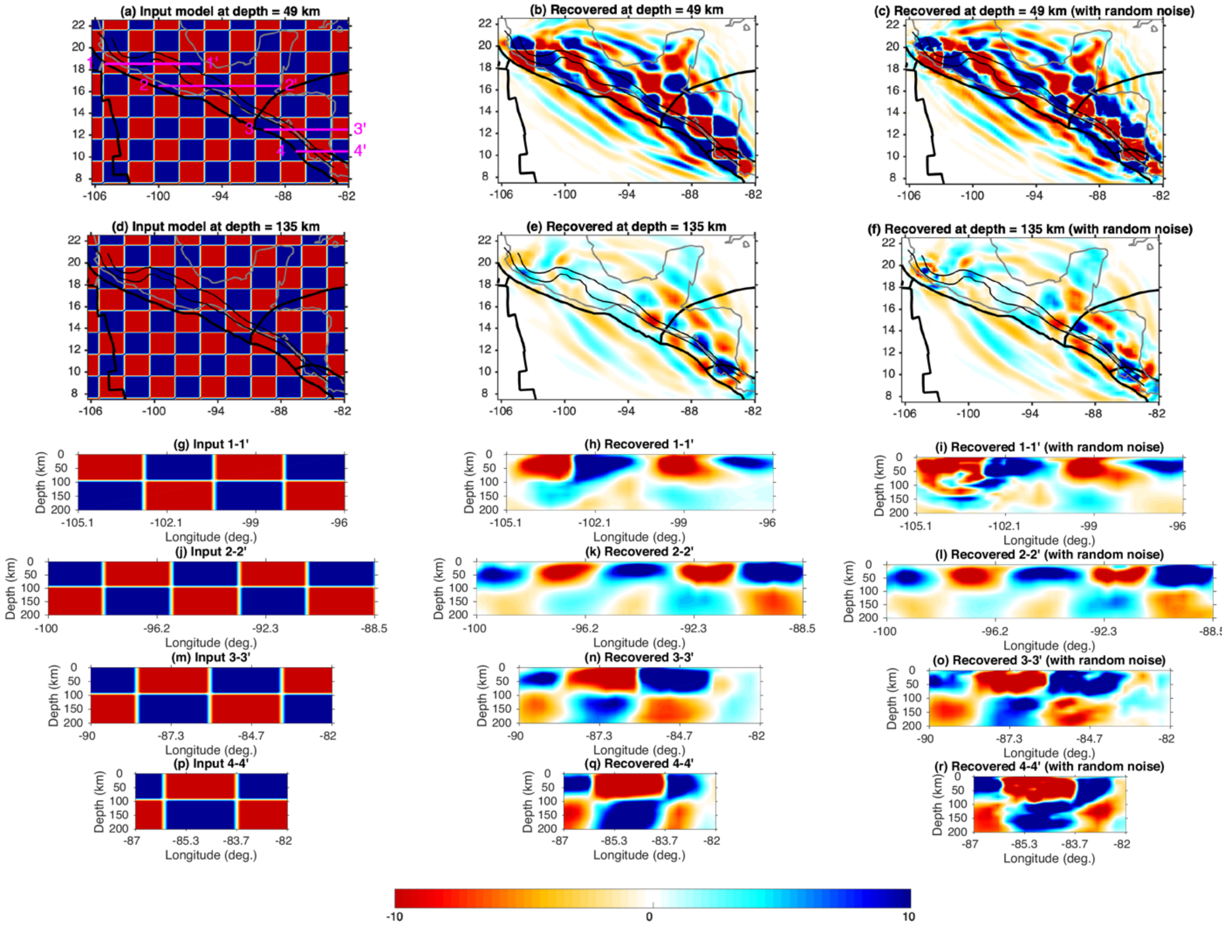

Figure S14. The three-dimensional checkerboard resolution tests at depths of 0-200 km. The horizontal dimension of the cells is 220 km. We set a sharp velocity boundary at the depth of 100 km. The velocity perturbation varies within a range of ±10%. The first column shows the checkerboard input model. The recovered models are displayed to the second (without random nose) and (with random nose) columns, correspondingly. The noise is based on the standard deviation of the phase delay measurements after the 3rd iteration.

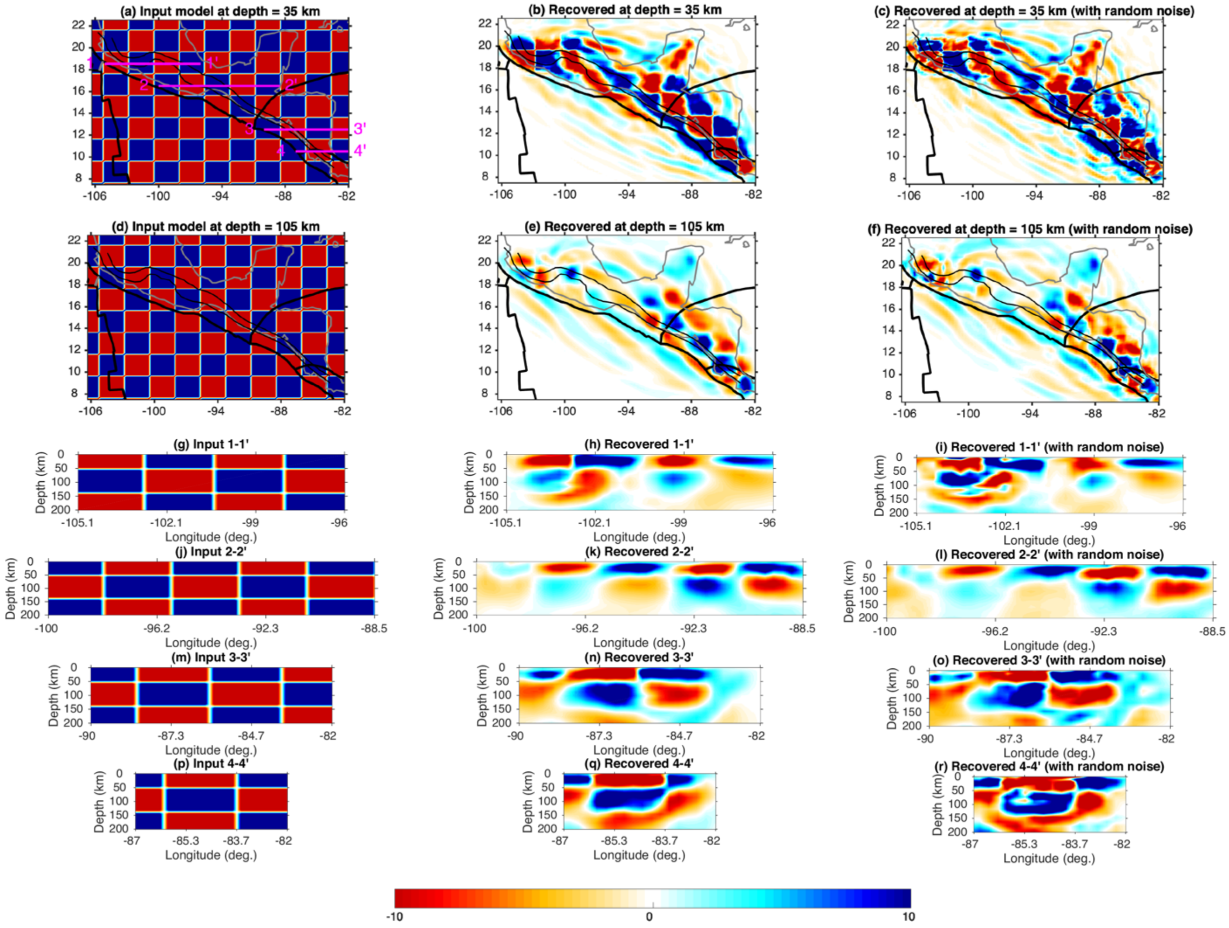

Figure S15. The three-dimensional checkerboard resolution tests at depths of 0-200 km. The horizontal dimension of the cells is 220 km. The two sharp velocity boundaries are at the depths of 50 km and 150 km, respectively. The velocity perturbation varies within a range of ±10%. The first column shows the checkerboard input model. The recovered models are displayed to the second (without random nose) and third (with random nose) columns, correspondingly. The noise is based on the standard deviation of the phase delay measurements after the 3rd iteration.

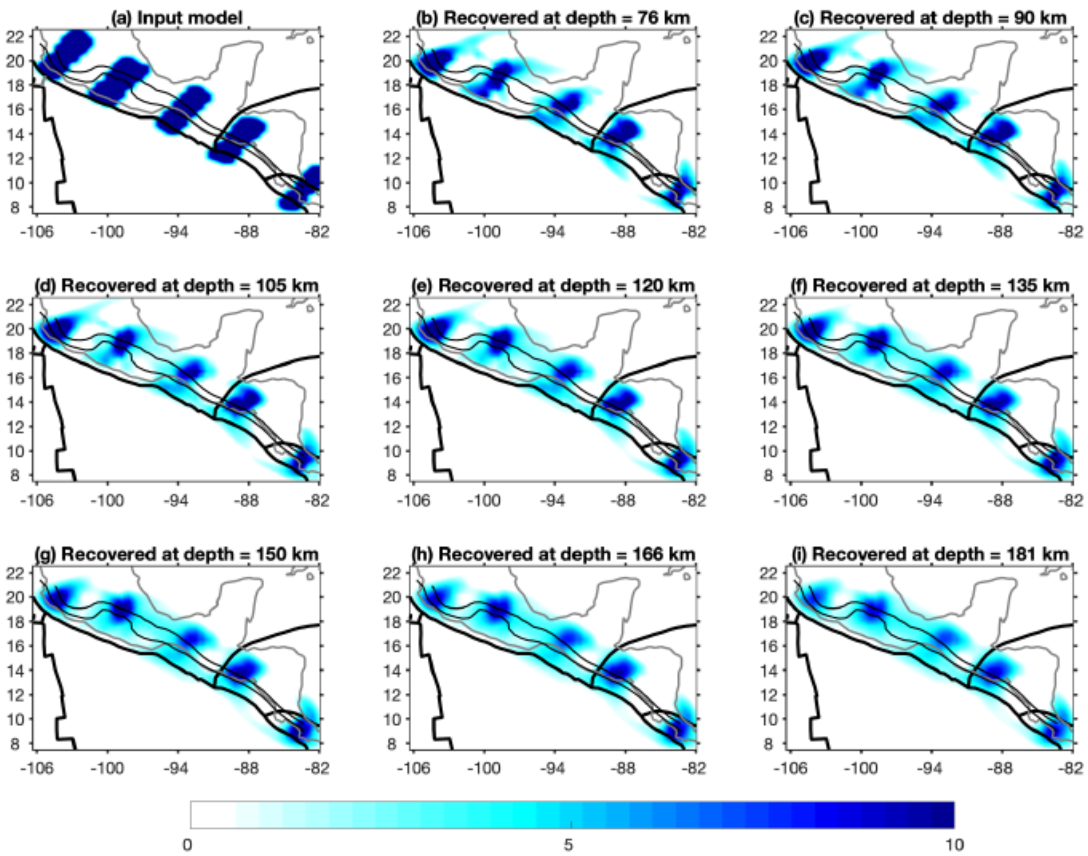

Figure S16. Model recovery test for along-strike smearing. (a) The input model includes five high-velocity anomalies oriented in the SW-NE direction, given a +10% velocity perturbation. (b)-(i) The recovered results of the input model at multiple depths.

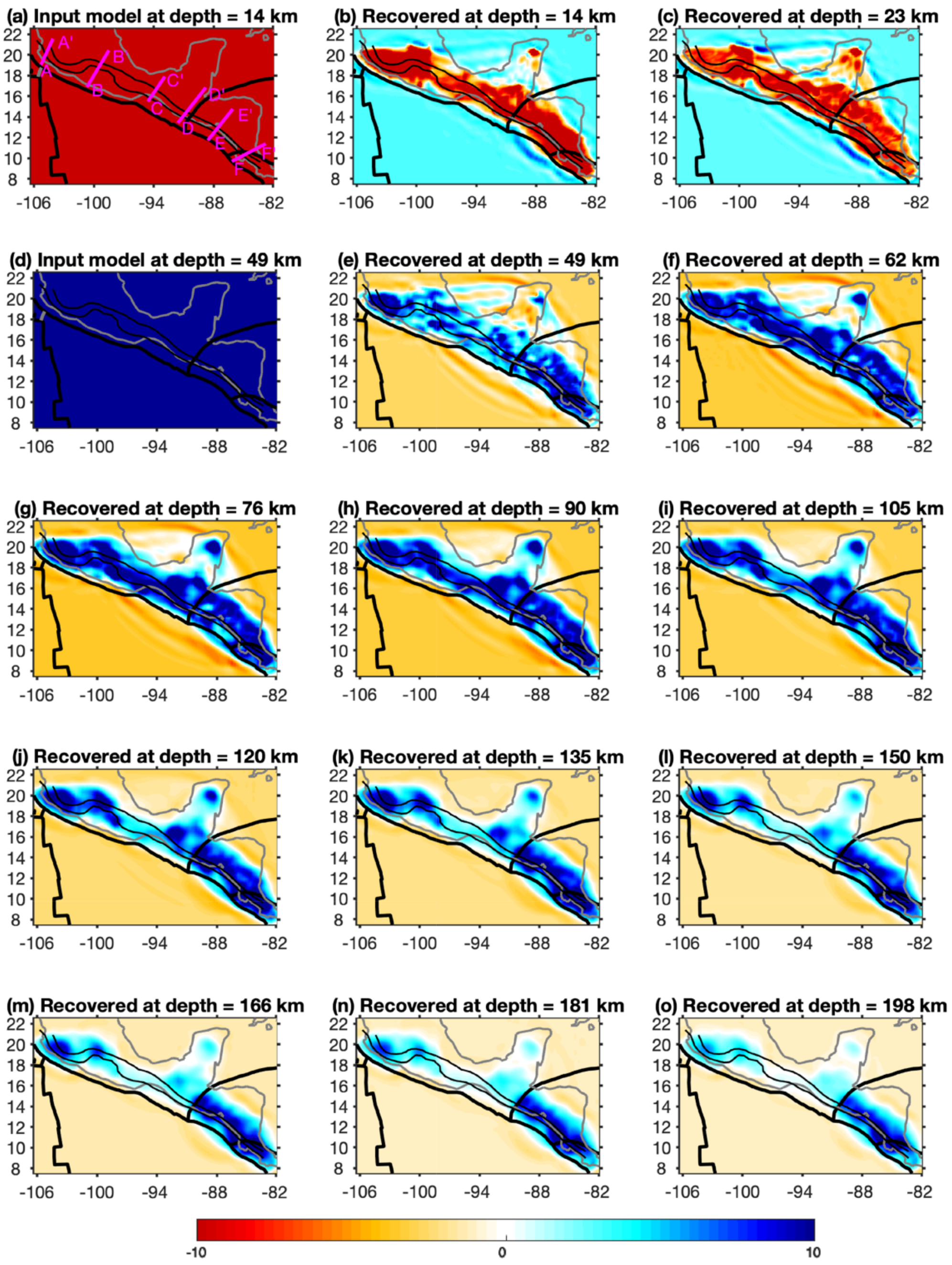

Figure S17. Model recovery test for a 200-km-thick lithosphere. The input model includes a -10% velocity perturbation at the depths of 0-40 km (a) and a +10% velocity perturbation at the depths of 40-200 km (d). (b)-(c) The recovered results of the crust. (e)-(o) The recovered results of the mantle lithosphere. The magenta lines in (a) show the profile locations in Fig. S18.

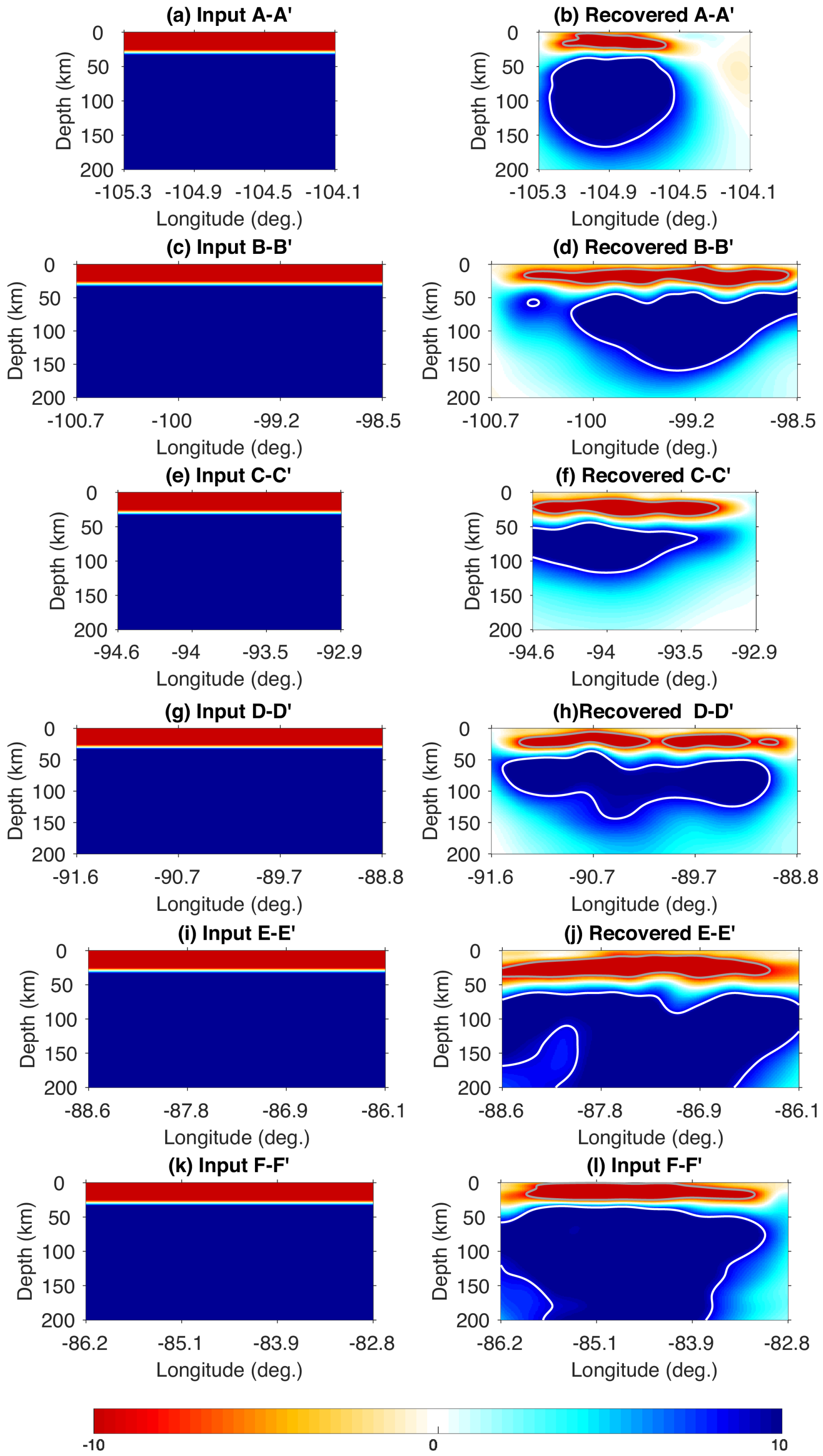

Figure S18. The vertical profiles of the model recovery test for a 200-km-thick lithosphere. The left column shows the vertical profiles for the input model. See the profile locations in Fig. S17. The recovered models are displayed to the right, correspondingly. The white lines represent the contour of the +7% velocity perturbation. The gray lines represent the contour of the -7% velocity perturbation.

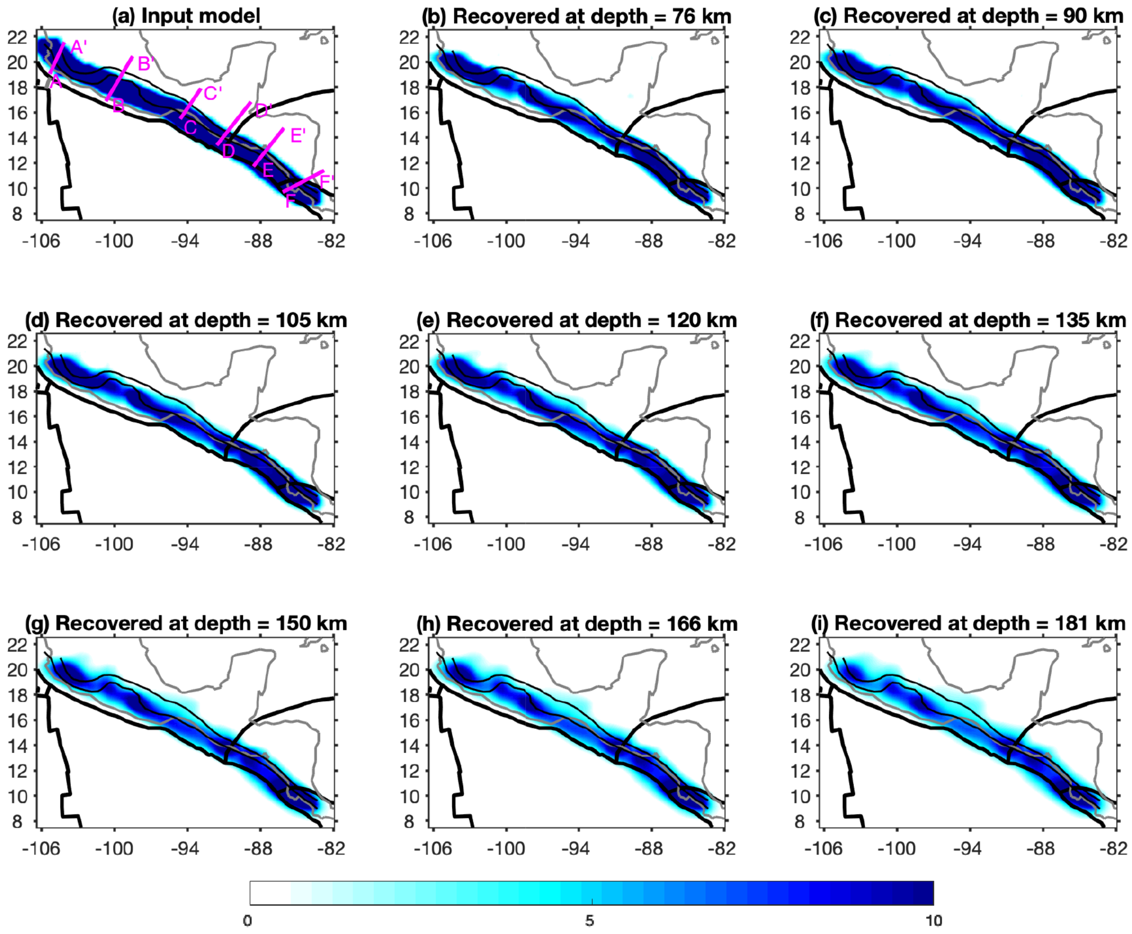

Figure S19. Model recovery test for a continuous slab. (a) The input model includes a +10% velocity perturbation for the oceanic lithosphere at the depths of 40-200 km. The width of the subducting slab is 150 km. (b)-(i) The recovered results of the oceanic lithosphere. The magenta lines in (a) show the profile locations in Fig. S20.

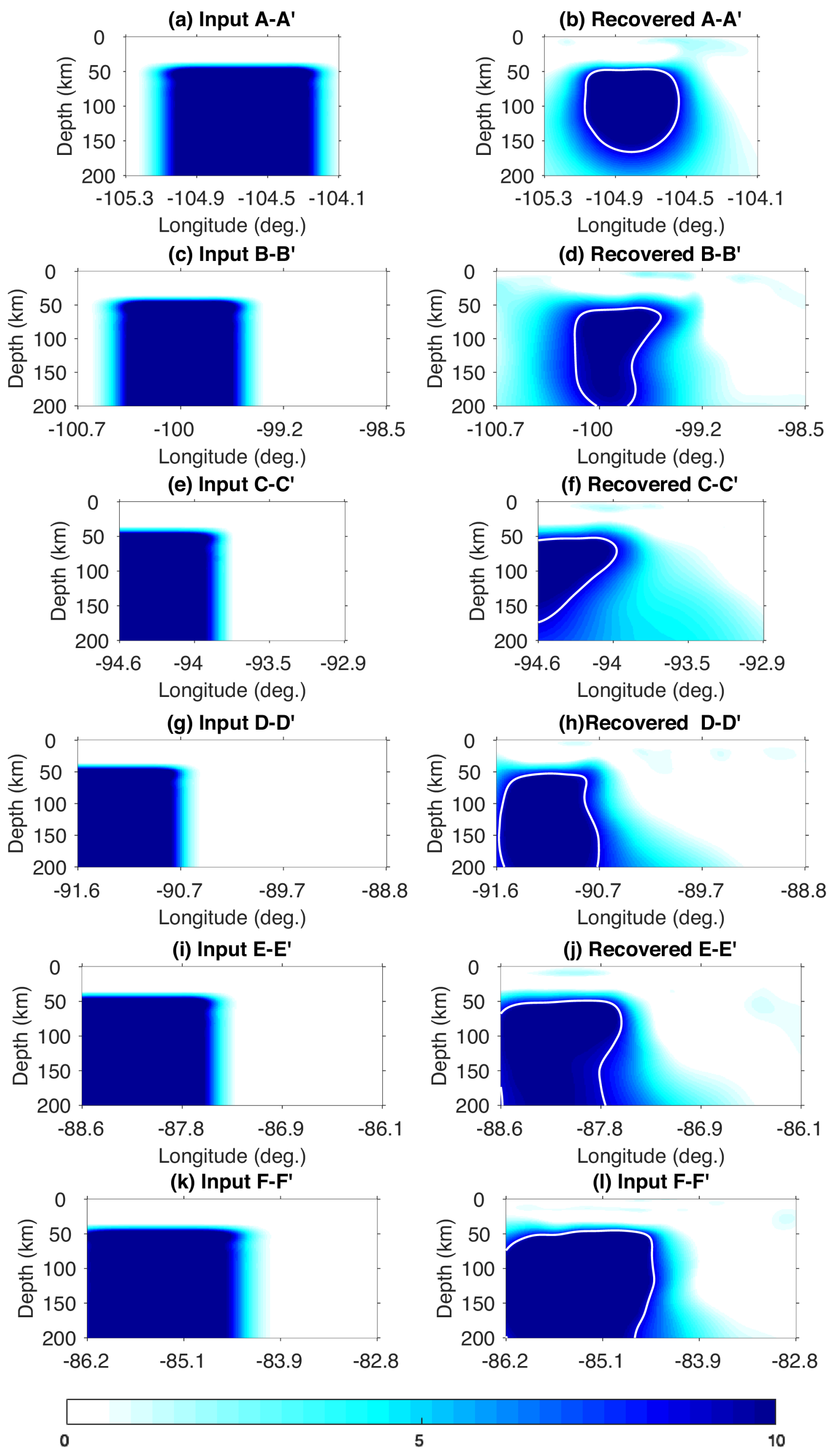

Figure S20. The vertical profiles of the model recovery test for a continuous slab. The left column shows the vertical profiles for the input model. See the profile locations in Fig. S19. The recovered models are displayed to the right, correspondingly. The white lines represent the contour of +7% velocity perturbation.

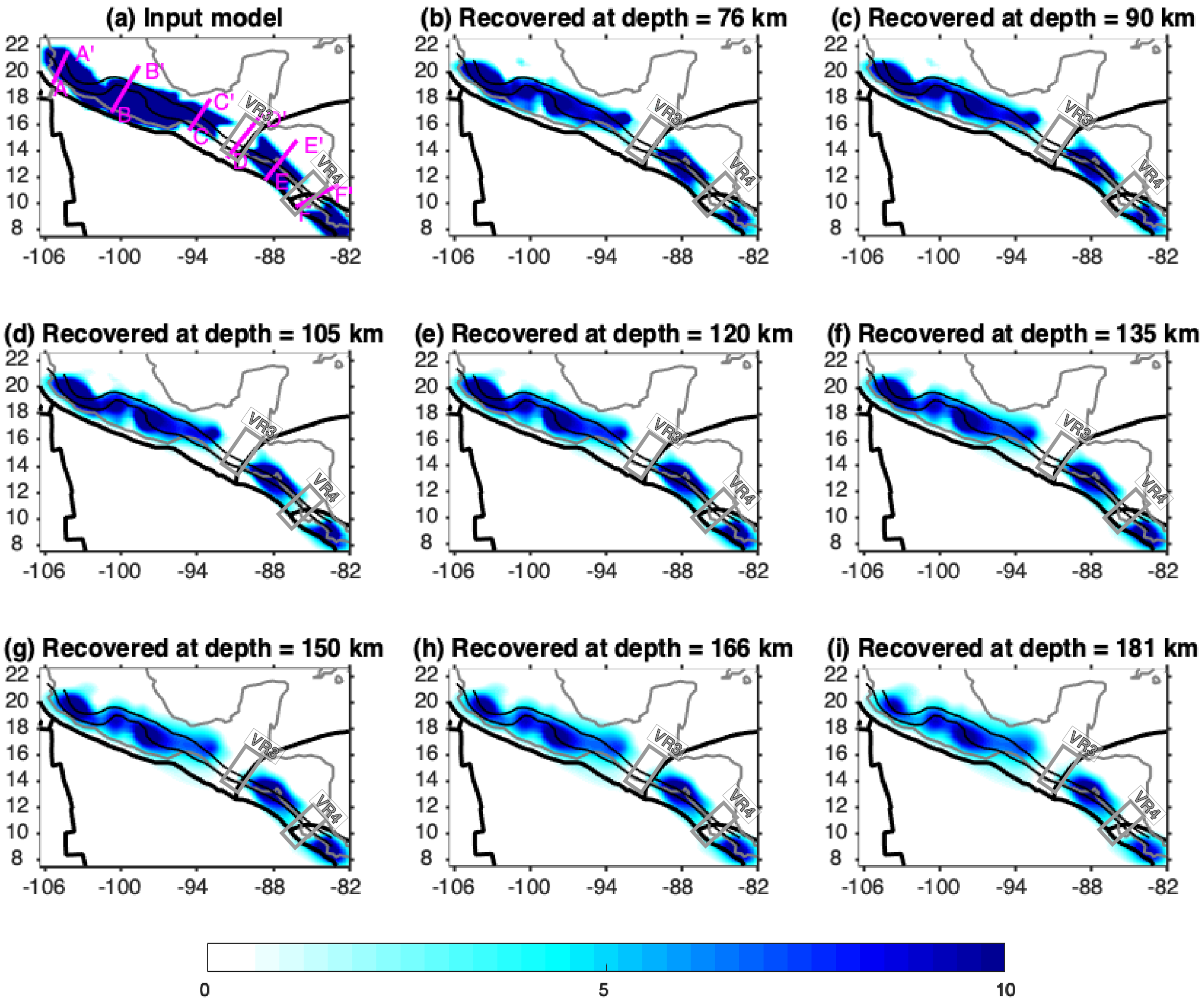

Figure S21. Model recovery test for slab segmentation along strike. (a) The input model includes a +10% velocity perturbation for the oceanic lithosphere. The subducting slab is continuous at longitudes of ~106°W - 91.5°W. The flat slab segment is at longitudes of ~102.5°W-95.5°W. The slab gaps are located near the NA-CB plate boundary and the CA-PA plate boundary. (b)-(i) The recovered results of the slab segmentation. The magenta lines in (a) show the profile locations in Fig. S22.

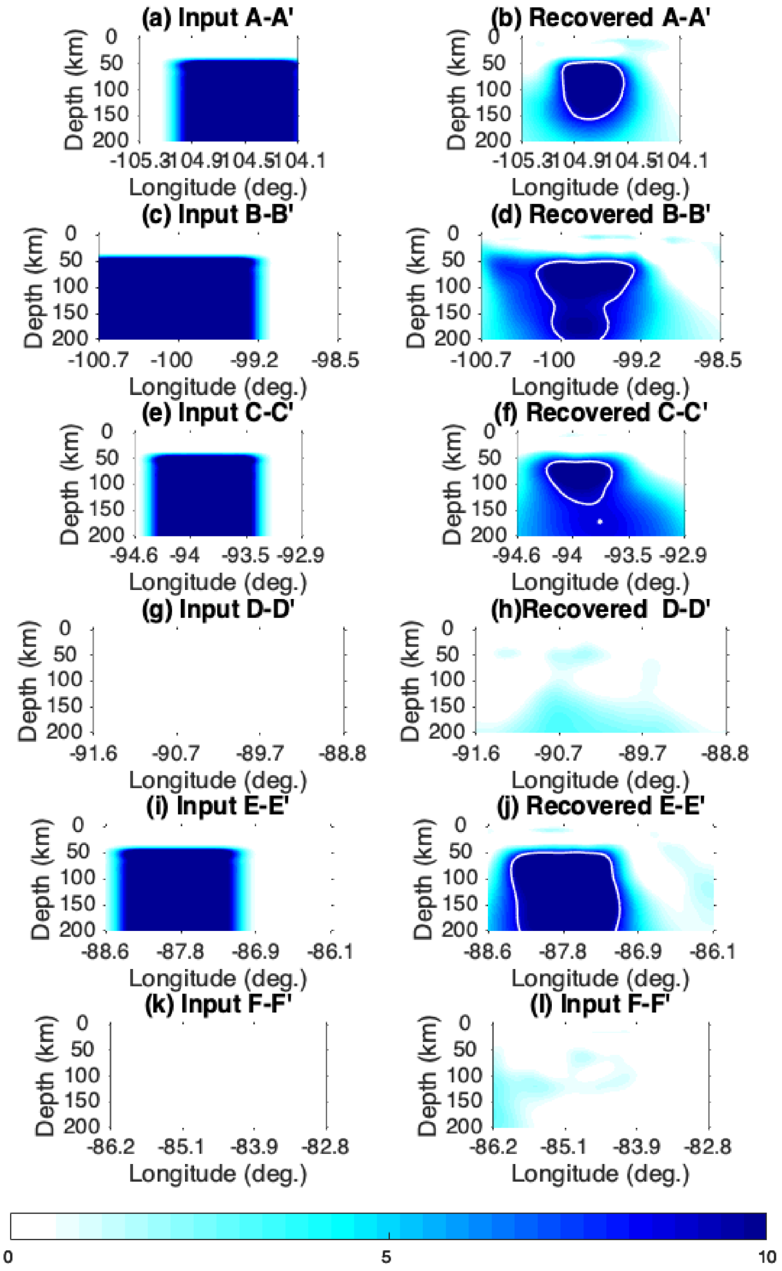

Figure S22. The vertical profiles of the model recovery test for slab segmentation. The left column shows the vertical profiles for the input model. See the profile locations in Fig. S21. The white lines represent the contour of +7% velocity perturbation. The gray lines represent the contour of -7% velocity perturbation. The recovered models are displayed to the right, correspondingly.

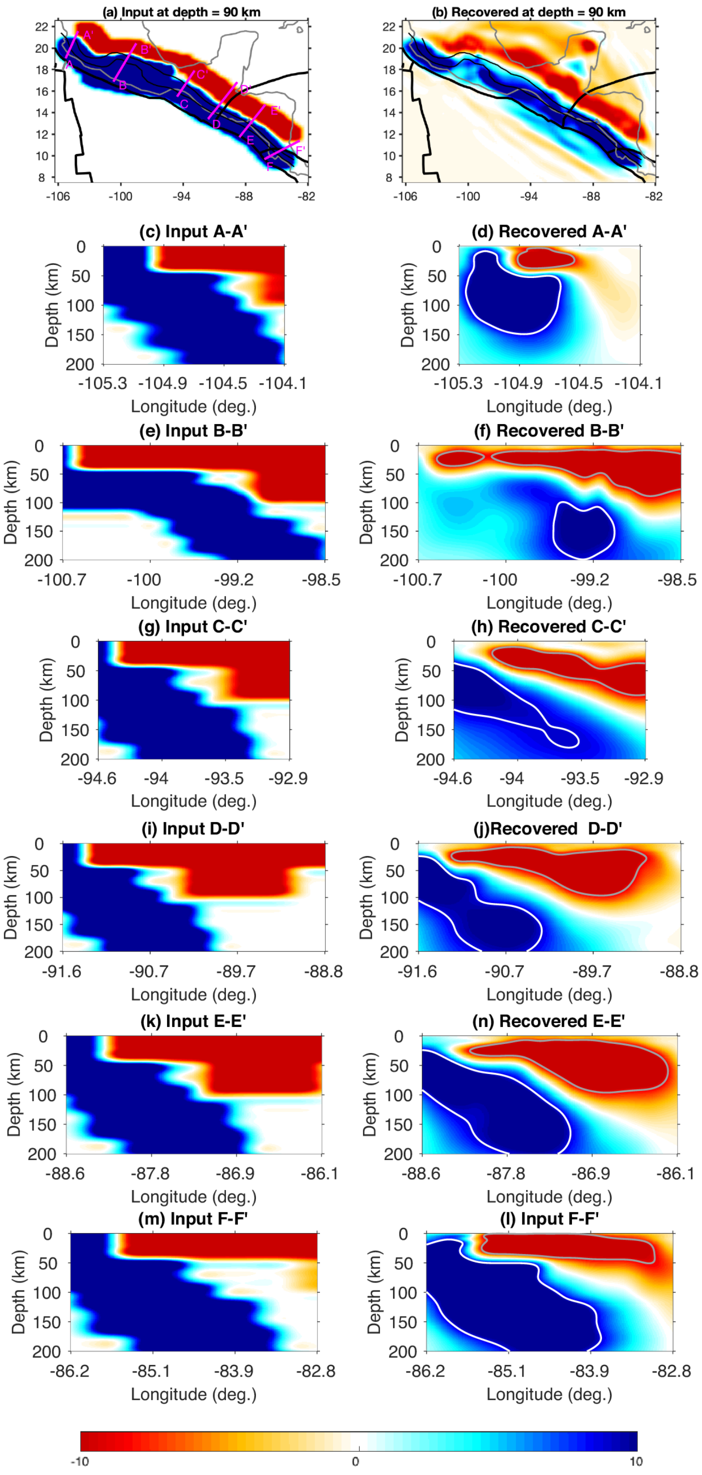

Figure S23. Model recovery test for the geometry of the subducting slab. The input model (left column) includes a +10% velocity perturbation for the slab and a -10% velocity perturbation for the continental crust and the mantle wedge. The recovered models are displayed to the right, correspondingly. The white lines represent the contour of +7% velocity perturbation. The gray lines represent the contour of -7% velocity perturbation.

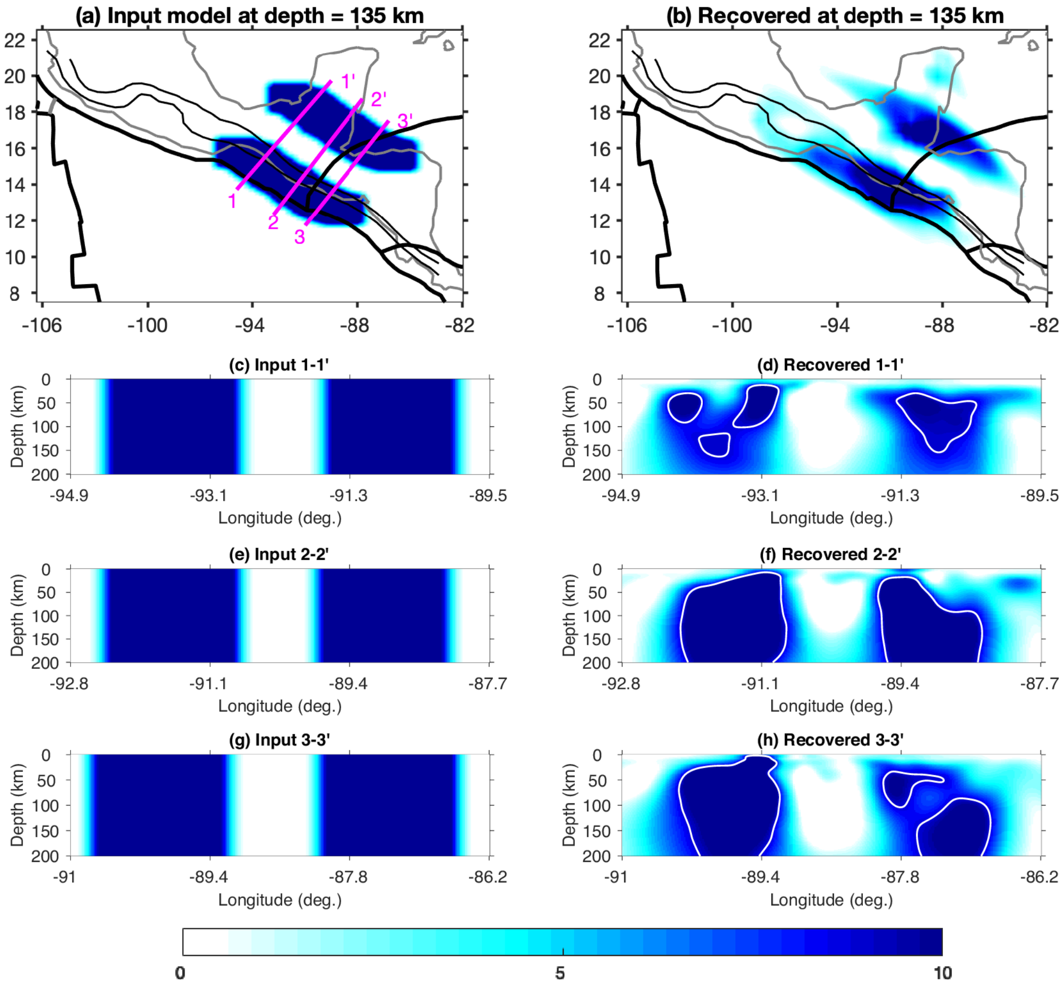

Figure S24. Model recovery tests for the central portion of the Middle American subduction system. The input model (left column) includes +10% velocity perturbations for the central Cocos slab and the subducted Yucatan slab, respectively. The recovered models are displayed to the right, correspondingly. The white lines represent the contour of +7% velocity perturbation.

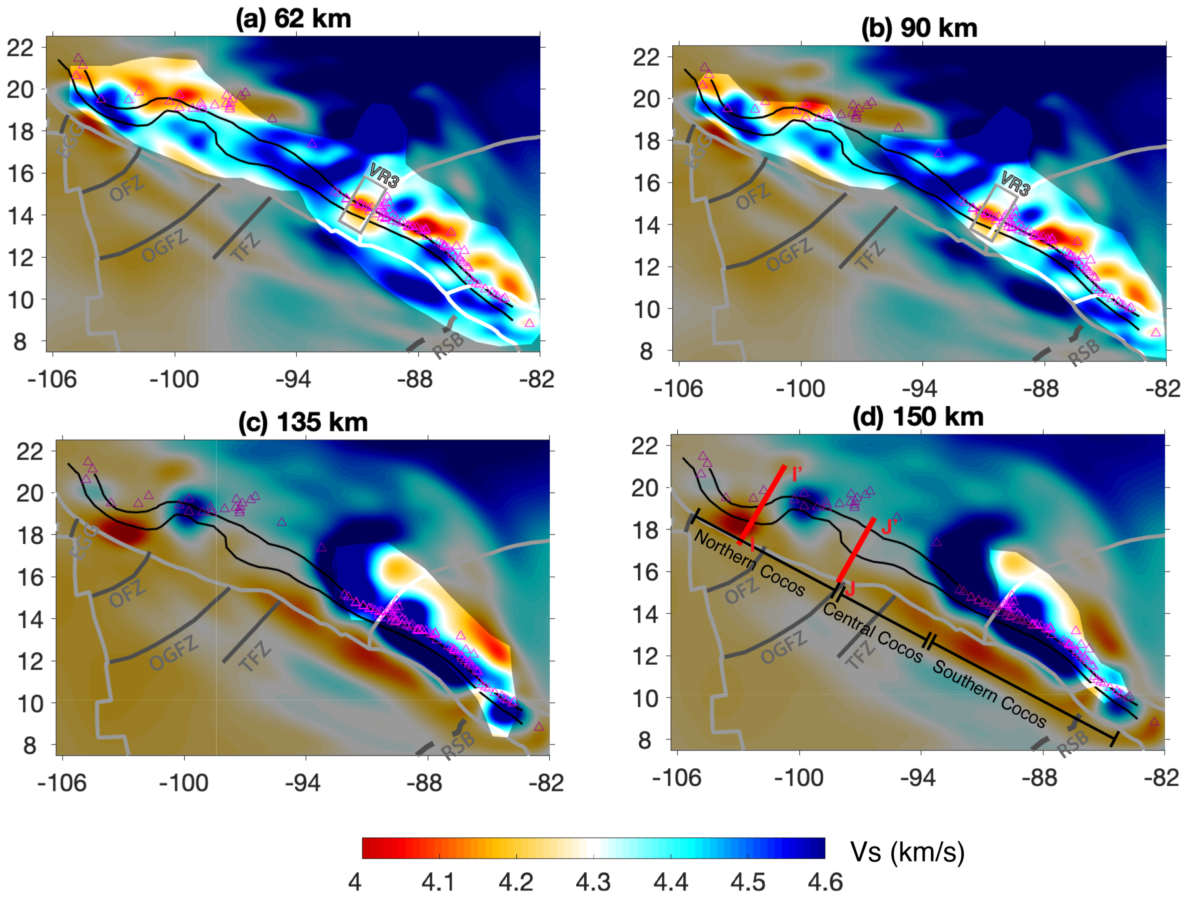

Figure S25. Shear-wave velocities at the depths of 62 km, 90 km, 135 km, and 150 km. The gray shaded areas mask the regions with low resolutions (i.e. less than 70% recovery of the velocity perturbation) based on the checkerboard resolution test in Fig. S12. The red lines in (d) mark the profile locations in Fig. S26. Other symbols are the same as in Fig. 2.

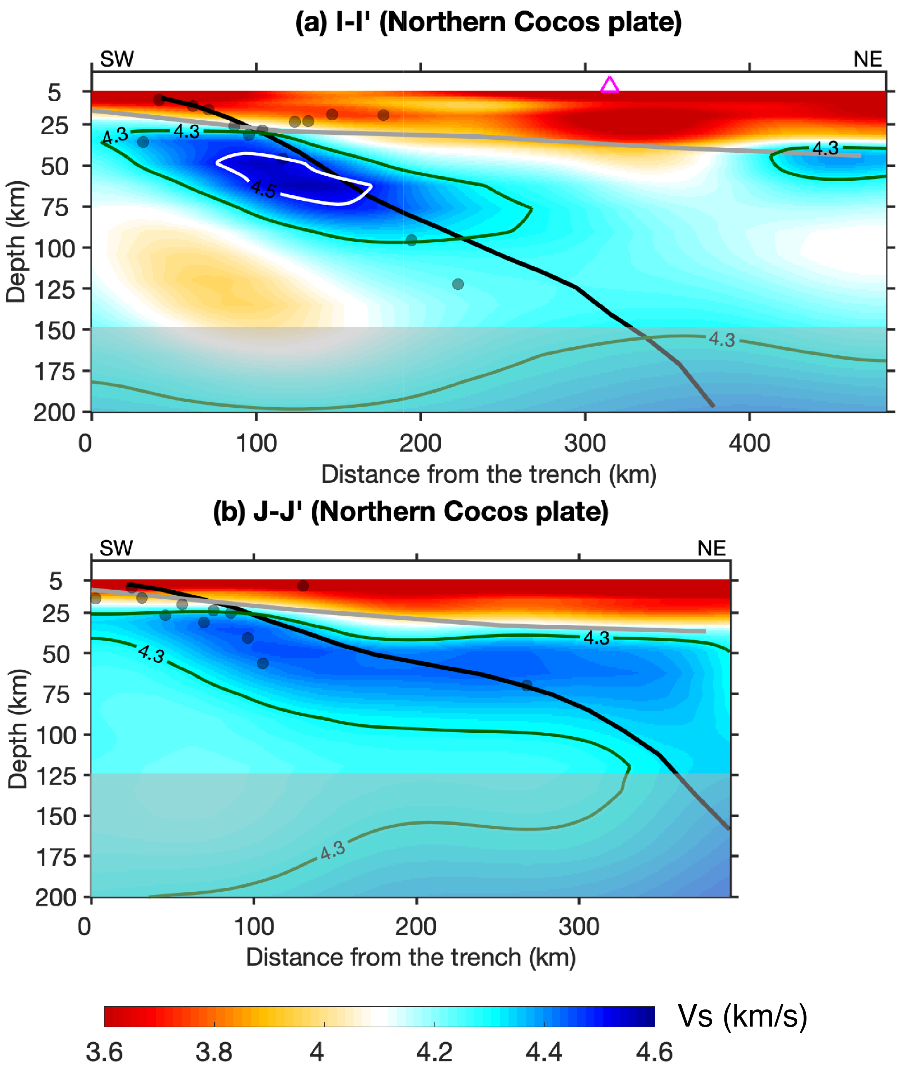

Figure S26. Vertical profiles of the tomographic model at depths of 5-200 km. The black lines represent the plate interface[1]. The gray lines represent the Moho depth extracted from CRUST 1.0[2]. The white lines represent the Vs = 4.5 km/s contour. There is no exaggeration of the vertical profiles. Other symbols are the same as in Fig. 3.

References

1. Hayes, G. P. *et al.* Slab2, a comprehensive subduction zone geometry model. *Science (80-.).* **362**, 58–61 (2018).
2. Laske, G., Masters, G., Ma, Z., Pasyanos, M. E. & Livermore, L. EGU2013-2658 Update on CRUST1.0 : A 1-degree Global Model of Earth's Crust. *Geophys. Res. Abstr.* **15**, Abstract EGU2013–2658 (2013).
